# Structural equation modeling for identifying the drivers of health-related quality of life improvement experienced by patients with migraine receiving eptinezumab

**DOI:** 10.1186/s10194-024-01752-z

**Published:** 2024-03-28

**Authors:** Linus Jönsson, Susanne F. Awad, Stephane A. Regnier, Brian Talon, Steven Kymes, Xin Ying Lee, Peter J. Goadsby

**Affiliations:** 1 Department for Neurobiology, Care Sciences and Society, Division of Neurogeriatrics, Karolinska Institutet, Solna, Sweden; 2 H. Lundbeck A/S, Copenhagen, Denmark; 3 Lundbeck LLC, Deerfield, IL USA; 4 NIHR King’s Clinical Research Facility and Headache Group, King’s College London, London, UK; 5 Department of Neurology, University of California, Los Angeles, CA USA

**Keywords:** Eptinezumab, Migraine, Treatment efficacy, Structural equation modeling

## Abstract

**Background:**

As new migraine therapies emerge, it is crucial for measures to capture the complexities of health-related quality of life (HRQoL) improvement beyond improvements in monthly migraine day (MMD) reduction. Investigations into the correlations between MMD reduction, symptom management, and HRQoL are lacking, particularly those that focus on improvements in canonical symptoms and improvement in patient-identified most-bothersome symptoms (PI-MBS), in patients treated with eptinezumab. This exploratory analysis identified efficacy measures mediating the effect of eptinezumab on HRQoL improvements in patients with migraine.

**Methods:**

Data from the DELIVER study of patients with 2–4 prior preventive migraine treatment failures (NCT04418765) were inputted to two structural equation models describing sources of HRQoL improvement via Migraine-Specific Quality-of-Life Questionnaire (MSQ) scores. A single latent variable was defined to represent HRQoL and describe the sources of HRQoL in DELIVER. One model included all migraine symptoms while the second model included the PI-MBS as the only migraine symptom. Mediating variables capturing different aspects of efficacy included MMDs, other canonical symptoms, and PI-MBS.

**Results:**

In the first model, reductions in MMDs and other canonical symptoms accounted for 35% (standardized effect size [SES] − 0.11) and 25% (SES − 0.08) of HRQoL improvement, respectively, with 41% (SES − 0.13) of improvement comprising “direct treatment effect,” i.e., unexplained by mediators. In the second model, substantial HRQoL improvement with eptinezumab (86%; SES − 0.26) is due to MMD reduction (17%; SES − 0.05) and change in PI-MBS (69%; SES − 0.21).

**Conclusions:**

Improvements in HRQoL experienced by patients treated with eptinezumab can be substantially explained by its effect on migraine frequency and PI-MBS. Therefore, in addition to MMD reduction, healthcare providers should discuss PI-MBS improvements, since this may impact HRQoL. Health technology policymakers should consider implications of these findings in economic evaluation, as they point to alternative measurement of quality-adjusted life years to capture fully treatment benefits in cost-utility analyses.

**Trial registration:**

ClinicalTrials.gov (Identifier: NCT04418765; EudraCT (Identifier: 2019–004497-25; URL: https://www.clinicaltrialsregister.eu/ctr-search/search?query=2019-004497-25).

**Graphical Abstract:**

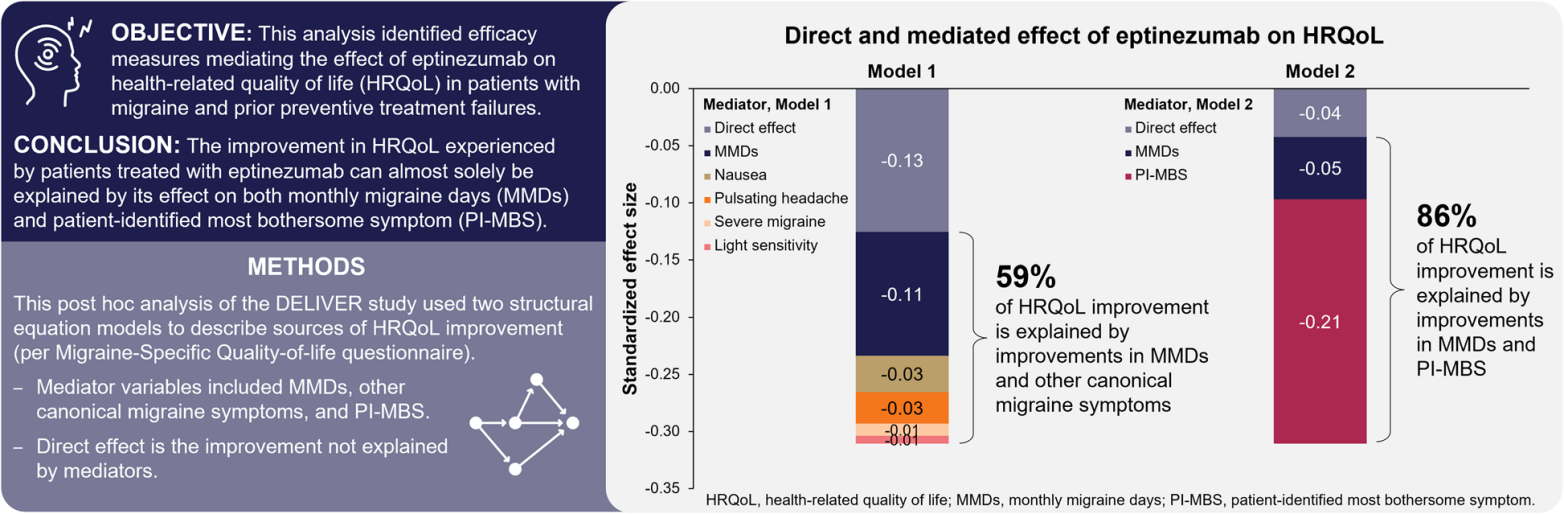

**Supplementary Information:**

The online version contains supplementary material available at 10.1186/s10194-024-01752-z.

## Introduction

Migraine is a disabling neurological disease [1] characterized by recurring, long-lasting attacks. It is ranked as the third most prevalent disorder in the world [2], resulting in substantial personal, societal, and financial burdens [2, 3]. In addition to headache-induced pain, patients with migraine also experience symptoms such as nausea, vomiting, and sensitivity to light and sound [2]. A multifaceted concept, health-related quality of life (HRQoL) refers to a specific type of quality of life that encompasses the physical, mental, and social aspects of a person’s health that may change in response to healthcare [4, 5]. In clinical trials, HRQoL can be assessed using a wide range of generalized or disease-specific instruments [6–9].

For a headache to be classified as migraine, it must have certain features: (a) unilateral location, pulsating quality, moderate or severe pain intensity, and/or aggravation by or causing avoidance of routine physical activity (≥ 2 of 4 symptoms), and (b) nausea and/or vomiting, or photophobia and phonophobia (≥ 1 of 2 symptoms) [2]. These are referred to as canonical symptoms; however, these symptoms do not comprise the full range of symptoms that an individual having a migraine may experience [10, 11]. Several clinical trials with eptinezumab have incorporated a patient-identified most bothersome symptom (PI-MBS) outcome measure that is not constrained to canonical symptoms [12–15]. Analysis of PI-MBS has underscored the heterogeneous nature of migraine, showing that there are at least 23 symptom types patients may consider most bothersome and that 16% of patients identify symptoms outside diagnostic criteria as their most bothersome [13].

As new migraine therapies emerge, measures that can capture the complexities of HRQoL improvement beyond improvements in monthly migraine day (MMD) reduction can help provide a more comprehensive picture of therapeutic benefits by helping patients, physicians, and policymakers to better understand those factors affected by treatment that drive improvement in quality of life, work productivity, and daily function. Eptinezumab, a humanized monoclonal antibody against the calcitonin gene-related peptide (CGRP) developed for the treatment of chronic and episodic migraine [16], was shown to be effective and well tolerated in clinical trials [12, 14, 15]. However, there have yet to be any investigations into the correlations between MMD reduction, symptom management, and HRQoL, particularly with a focus on improvements in canonical symptoms and improvement in PI-MBS, in patients treated with eptinezumab.

The overall aim of this post hoc analysis of the DELIVER clinical trial [15] was to conduct a mediation analysis that could identify the impact of eptinezumab on HRQoL through various mediators of treatment efficacy (i.e., changes in MMDs, migraine severity, canonical symptoms, and PI-MBS) and compare it to the direct (i.e., unexplained by the above efficacy variables) effect of eptinezumab on HRQoL as measured by the Migraine-Specific Quality of Life Questionnaire (MSQ).

## Methods

### Study population and design

Data for this post hoc analysis were from the DELIVER clinical trial (NCT04418765)—a multinational, phase 3b, randomized, double-blind, placebo-controlled study that explored the safety and efficacy of eptinezumab treatment in patients with migraine and 2–4 prior preventive migraine treatment failures [15]. Conducted from June 1, 2020, to October 7, 2021, the placebo-controlled portion of DELIVER tracked patient change from baseline in MMDs over 24 weeks, after receiving up to 2 doses of eptinezumab (100 mg or 300 mg) or placebo [15]. Eptinezumab administration via intravenous infusion occurred at baseline (day 0) and Week 12 [15]. The primary endpoint was the mean change from baseline in MMDs over Weeks 1–12; secondary endpoints were PI-MBS, Patient Global Impression of Change (PGIC), and changes in the frequency of canonical symptoms [2].

For each headache episode throughout the study, patients were to complete an electronic diary recording the headache episode start/stop date/times and the characteristics used for classifying headache episodes as migraine attacks (i.e., canonical symptoms). Based on International Headache Society guidelines [7], migraine days were study days that met one of the following criteria: the patient had a headache that lasted ≥4 hours and met International Classification of Headache Disorders, 3rd edition (ICHD-3) criteria C and D for migraine without aura; the patient had a 30-minute or longer headache and had migraine with aura; the patient had a 30-minute or longer headache that met two of the three ICHD-3 criteria B, C, and D for migraine without aura (probable migraine); or the patient believed they had a migraine and thus took acute migraine medication [15]. Migraine attacks are single continuous events and can last more than 24 hours. To determine if headache episodes were migraine attacks, the headache diary asked patients whether they experienced the canonical symptoms of migraine, as defined above [2].

The PI-MBS is a patient-reported outcome measure in which patients are asked to describe their most bothersome symptom at the baseline visit, which is then categorized by investigators into eight predefined symptom classes (nausea, vomiting, sensitivity to light, sensitivity to sound, mental cloudiness, fatigue, pain with activity, mood changes) or “other” with free text [17]. At 4- to 12-week intervals following identification, improvements in the PI-MBS were rated on a 7-point scale identical to the PGIC scale (“very much worse” [− 3] to “very much improved” [+ 3]). Data from PROMISE-2 showed that improvement scores on the PI-MBS were highly correlated with PGIC scores, and more correlated compared to PGIC scores and the primary endpoint, changes in MMDs [13].

Quality of life was assessed in the DELIVER trial with two instruments: the MSQ and the 6-item Headache Impact Test (HIT-6) [8, 9].

### Analytical framework

We conducted the mediation analysis with structural equation modeling (SEM), an analytic tool that can consider the inclusion of variables that are not measured directly, but measured through their observable effects, and allows assessment of causal relationships and mediating factors [18, 19]. SEM can be thought of as combining path analysis, which aims at discerning causal pathways, with latent variables. The SEM models in this work had three components: a latent variable (HRQoL) and its related measurements, a treatment effect, and a set of variables mediating the treatment effect on HRQoL. Variables included in this analysis, including the change from baseline in MMDs, were reported monthly for the first 6 months of the trial (Weeks 1–4, Weeks 5–8, Weeks 9–12, Weeks 13–16, Weeks 17–20, and Weeks 21–24), meaning each patient had up to six values post baseline.

### Latent variable identification

SEM involved constructing, in this analysis, an HRQoL latent variable, which can be thought of as the study outcome. The rationale for using a latent variable is that HRQoL cannot be directly observed and measured; however, it can be approximated by various measures such as patient-reported outcomes [4, 20]. We started by defining a single latent variable to represent HRQoL and included all individual items of the quality-of-life scales captured in the DELIVER trial as measures of this latent variable. This first model (Model A) included the 14 individual items of the MSQ and the HIT-6. We compared the fit of this model with more restricted versions, Model B including the individual items of the MSQ (excluding the HIT-6), and Model C combining the MSQ items into three domain scores: Role Function–Restrictive [RR], Role Function–Preventive [RP], and Emotional Function [EF] [8]. The latter model was found to have the best fit to data; results are presented in the Online Supplemental Material (see Model Details).

### Mediation analysis

The mediating variables were variables that can potentially affect the outcome (i.e., improvement in the HRQoL latent variable) and may be affected by eptinezumab. Potential mediating variables considered in this analysis to explain HRQoL improvement included changes in: MMDs; monthly migraine attacks; proportion of severe migraine attacks; and proportion of migraine attacks with the following canonical symptoms: nausea, vomiting, light sensitivity, aura, aggravation by physical activity, throbbing/pulsating quality, one-sidedness, and sound sensitivity; and PI-MBS. Models with migraine frequency and PGIC instead of PI-MBS as mediator were also analyzed and found to have satisfactory fit statistics, but PI-MBS became the focus of this analysis given its high correlation with PGIC [13] while also encompassing improvement in a symptom specific to migraine.

Initially, all canonical symptoms per ICHD-3 diagnostic criteria [2] for migraine were identified as potential mediators, with the exception of “moderate or severe pain intensity,” which was limited to severe pain for these analyses and is labeled separately. A backwards elimination method (Supplemental Table 1) was used to identify mediators with *P*-values less than 0.05 for the association with HRQoL (for Models D, E, F, and G, see Model Details in Online Supplemental Material).

**Fig. 1:**
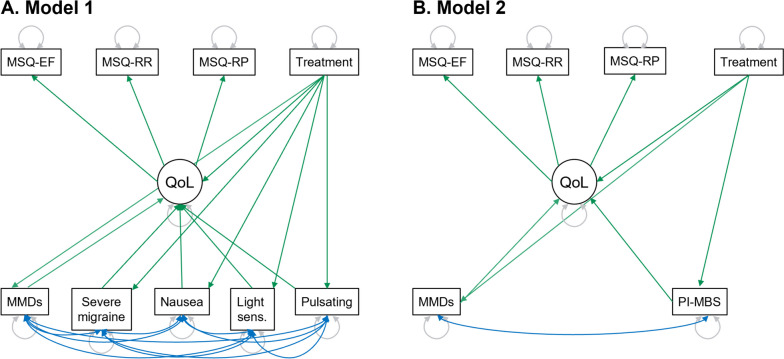
Structural equation diagrams for (**A**) Model 1 and (**B**) Model 2 Shapes: circle = latent variable; square/rectangle = measured variables. Color arrows: green arrows = paths (between variables); blue two-way arrows (between variables) = covariances; gray twoway arrows (variable to itself) = variances Abbreviations: MMDs, monthly migraine days; MSQ, Migraine-Specific Quality of Life Questionnaire; MSQ-EF, MSQ Emotional Function; MSQ-RP, MSQ Role Function-Preventive; MSQ-RR, MSQ Role Function-Restrictive; PI-MBS, patient-identified most bothersome symptom; QoL, (health-related) quality of life; Sens., sensitivity

These mediators were then included in the final Model 1. Thus, Model 1 (Fig. 1A) variables were: change from baseline in MMDs and changes from baseline in the percentage of monthly migraine attacks with severe pain intensity, nausea, pulsating/throbbing quality, and light sensitivity.

Given the hypothesis that HRQoL improvement would likely not be fully explained by Model 1, a second model was developed to include a new mediator representing migraine symptoms, i.e., the change from baseline in PI-MBS. Hence, Model 2 mediating variables were change from baseline in MMDs and change from baseline in PI-MBS (Fig. 1B). Since PI-MBS highlights the migraine-specific symptom that bothers the patient the most (and any improvement in that symptom by definition), all canonical symptoms included in Model 1 were excluded in Model 2. Predefined symptom categories for PI-MBS at baseline included pain with activity, fatigue, nausea, mental cloudiness, sensitivity to light, sensitivity to sound, vomiting, mood changes, and other (with free text) [17]. Because improvements in MMD and PI-MBS and between MMD and canonical symptoms could be correlated, both models allowed correlation between those variables (Fig. 1A and B).

In Models 1 and 2, eptinezumab treatment affected the latent variable (improvement in HRQoL) indirectly through several mediating variables capturing different aspects of treatment efficacy as well as directly (i.e., not otherwise explained by the efficacy mediators explored). The total effect of eptinezumab is the sum of the direct (coefficient of the effect on eptinezumab on latent variable) and indirect effects (multiplying the coefficient of the effect on eptinezumab on mediators by the coefficient of the effect of mediators on latent variable). The analyses were conducted using the average change from baseline during the double-blind period of DELIVER (Weeks 1–24). MMDs and canonical symptoms were analyzed monthly in the DELIVER double-blind period; thus, up to six data points per individual contributed to the average change over Weeks 1–24 in MMDs used in the model. However, PI-MBS and MSQ were captured at Weeks 12 and 24; thus, up to two measurements per individual contributed to the data. No imputations were run for missing PI-MBS, MSQ, or canonical symptoms data as the level of missingness was low (2.5% at 12 weeks and 6.1% at 24 weeks for PI-MBS).

### Model estimation

Models can only be estimated if they are identified, i.e., there is a sufficient number of observed variables in relation to parameters to be estimated. The number of free parameters to be estimated cannot exceed the number of observed variances/covariances, calculated as p*(p + 1)/2 where p is the number of observed variables. After confirming that models were identified, all models were estimated using maximum likelihood, with the nlminb optimizer. Goodness of fit of the model was evaluated based on the chi-square, comparative fit index (CFI), root mean square error of approximation (RMSEA), and standardized root-mean square residual (SRMR). An acceptable goodness of fit was defined as χ^2^/df (degree of freedom) < 2.0, CFI > 0.9, RMSEA < 0.05, and SRMR < 0.08 [21].

### Software

Analyses were conducted on pooled data from all DELIVER study visits during the double-blind portion of the trial (Weeks 1–24). SEM analysis was completed using the LAVAAN package (0.6–16) for R version 4.3.1.

## Results

### Study population

Table 1 presents patient demographics at baseline.

**Table 1:** Description of the patient population at baseline

	Placebo	Combined (eptinezumab 100 and 300 mg)	All treatment groups
N	299	593	892
Age, mean (SD)	43.8 (10.8)	43.8 (10.5)	43.8 (10.6)
Female, n (%)	264 (88.3)	538 (90.7)	802 (89.9)
Current migraine diagnosis, n (%)			
Chronic migraine	125 (41.8)	230 (38.8)	355 (39.8)
Episodic migraine	174 (58.2)	363 (61.2)	537 (60.2)
Duration of diagnosis, years, mean (SD)			
Chronic migraine	11.0 (10.9)	11.7 (10.8)	11.4 (10.8)
Episodic migraine	17.4 (12.1)	16.2 (11.2)	16.6 (11.5)
HRQoL measure scores			
MSQ-RR, mean (SD)	35.1 (17.1)	35.7 (16.8)	35.5 (17.0)
MSQ-RP, mean (SD)	50.5 (22.1)	50.6 (21.4)	50.6 (21.6)
MSQ-EF, mean (SD)	48.4 (26.6)	49.5 (24.2)	49.1 (25.1)
HIT-6, mean (SD)	66.2 (4.4)	66.5 (4.6)	66.4 (4.5)
MMDs, mean (SD)	13.9 (5.7)	13.8 (5.5)	13.8 (5.6)

*HIT-6* 6-item Headache Impact Test: *HRQoL* health-related quality of life: *MMDs* monthly migraine days: *MSQ* Migraine-Specific Quality of Life Questionnaire: *MSQ-EF* MSQ Emotional Function: *MSQ-RP* MSQ Role Function-Preventive: *MSQ-RR* MSQ Role Function-Restrictive: *SD* standard deviation

#### Final models

The fitted parameters of Models 1 and 2 are presented in Table 2. We standardized all parameter estimates, for observed as well as latent variables. Standardization was achieved by rescaling the raw parameter estimate by the ratio of the standard deviation of x over the standard deviation of y. In both models, the coefficients for the measures of the latent variable (MSQ domains) were significant and with standardized values of similar magnitude for the domains (around 0.8–0.9). In Model 1, the most important determinants of HRQoL scores were MMDs followed by migraine severity and nausea, in terms of the magnitude of standardized coefficients. In Model 2, PI-MBS was by far the most important variable determining HRQoL, followed by MMDs. Treatment significantly reduced all symptoms and migraine frequency in both models.

**Table 2:** Estimated parameters of Models 1 and 2

			Model 1					Model 2				
LHS		RHS	Estimate	SE	Z-score	p-value	SEst.	Estimate	SE	Z-score	*p*-value	SEst.
HRQoL	=~	MSQ-EF	1.000	0.000			0.784	1.000	0.000			0.781
HRQoL	=~	MSQ-RP	1.012	0.036	27.881	< 0.001	0.878	1.005	0.036	27.579	< 0.001	0.869
HRQoL	=~	MSQ-RR	1.057	0.037	28.865	< 0.001	0.921	1.072	0.037	29.193	< 0.001	0.931
HRQoL	~	PI-MBS						−9.322	0.650	−14.350	< 0.001	−0.538
HRQoL	~	MMDs	−1.446	0.135	−10.716	< 0.001	− 0.361	− 0.718	0.134	−5.373	< 0.001	−0.180
HRQoL	~	Nausea	−9.728	2.410	−4.036	< 0.001	−0.143					
HRQoL	~	Severe migraine	−0.113	0.026	−4.389	< 0.001	−0.151					
HRQoL	~	Pulsating/throbbing	−5.869	2.803	−2.094	0.036	−0.067					
HRQoL	~	Light sensitivity	−5.592	2.600	−2.151	0.031	−0.072					
HRQoL	~	Treatment (direct effect)	5.111	1.294	3.950	< 0.001	0.127	1.667	1.224	1.362	0.173	0.041
PI-MBS	~	Treatment (direct effect)						−0.932	0.074	−12.646	< 0.001	−0.401
MMDs	~	Treatment (direct effect)	−3.030	0.332	−9.116	< 0.001	−0.301	−3.030	0.332	−9.116	< 0.001	−0.301
Nausea	~	Treatment (direct effect)	−0.114	0.020	−5.643	< 0.001	−0.192					
Severe migraine	~	Treatment (direct effect)	−11.117	1.826	−6.089	< 0.001	−0.206					
Pulsating/throbbing	~	Treatment (direct effect)	−0.049	0.016	−3.081	0.002	−0.106					
Light sensitivity	~	Treatment (direct effect)	−0.078	0.018	−4.378	< 0.001	−0.150					

= ~ indicates measurement of a latent variable, ~ indicates regression

*HRQoL* health-related quality of life: *LHS* left-hand side variable: *MMDs* monthly migraine days: *MSQ* Migraine-Specific Quality of Life Questionnaire: *MSQ-EF* MSQ Emotional Function: *MSQ-RP* MSQ Role Function-Preventive: *MSQ-RR* MSQ Role Function-Restrictive: *PI-MBS* patient-identified most bothersome symptom: *RHS* right-hand side variable: *SE* standard error: *SEst* standard estimate

### Mediation analysis

Table 3 presents the analysis of direct and indirect effects of treatment on HRQoL. In Model 1, the total standardized effect size was 0.312, out of which 0.185 was an indirect effect mediated through effects on MMDs and canonical symptoms. There was a large remaining direct effect that could not be explained through the Model 1 mediators, despite the inclusion of several canonical symptoms. The contribution of each factor to HRQoL, by percentage, was as follows: the direct effect of eptinezumab (41%), changes in MMDs (35%), and changes in percentage of migraine attacks with severe pain intensity (10%), with nausea (9%), with light sensitivity (3%), and with presence of pulsating/throbbing headache pain (2%). Notably, the 35% contribution of MMD reduction meant that MMDs only explained approximately one-third of HRQoL improvement. The χ^2^/df of 1.4, SRMR of 0.009, RMSEA of 0.023, and CFI of 0.998 statistics all indicated a satisfactory fit for Model 1 (Supplemental Table 2).

**Table 3:** Mediation analysis

	Model 1						Model 2					
	Estimate	SE	Z-score	p-value	SEst.	% of total effect	Estimate	SE	Z-score	*p*-value	SEst.	% of total effect
HRQoL direct effect	5.111	1.294	3.950	< 0.001	0.127	41	1.667	1.224	1.362	0.173	0.041	
HRQoL indirect effect	7.470	0.858	8.710	< 0.001	0.185	–	10.860	1.016	10.689	< 0.001	0.270	
HRQoL_PI-MBS						–	8.686	0.915	9.488	< 0.001	0.216	69
HRQoL_MMDs	4.381	0.631	6.943	< 0.001	0.109	35	2.175	0.470	4.629	< 0.001	0.054	17
HRQoL_Nausea	1.108	0.337	3.283	0.001	0.027	9						
HRQoL_Severe migraine	1.259	0.353	3.561	< 0.001	0.031	10						
HRQoL_Pulsating/throbbing	0.286	0.165	1.732	0.083	0.007	2						
HRQoL_Light sensitivity	0.437	0.226	1.930	0.054	0.011	3						
HRQoL total effect	12.581	1.438	8.749	< 0.001	0.312	–	12.527	1.430	8.757	< 0.001	0.312	–

*HRQoL* health-related quality of life: *MMDs* monthly migraine days: *PI-MBS* patient-identified most bothersome symptom: *SE* standard error: *SEst* standard estimate

**Fig. 2:**
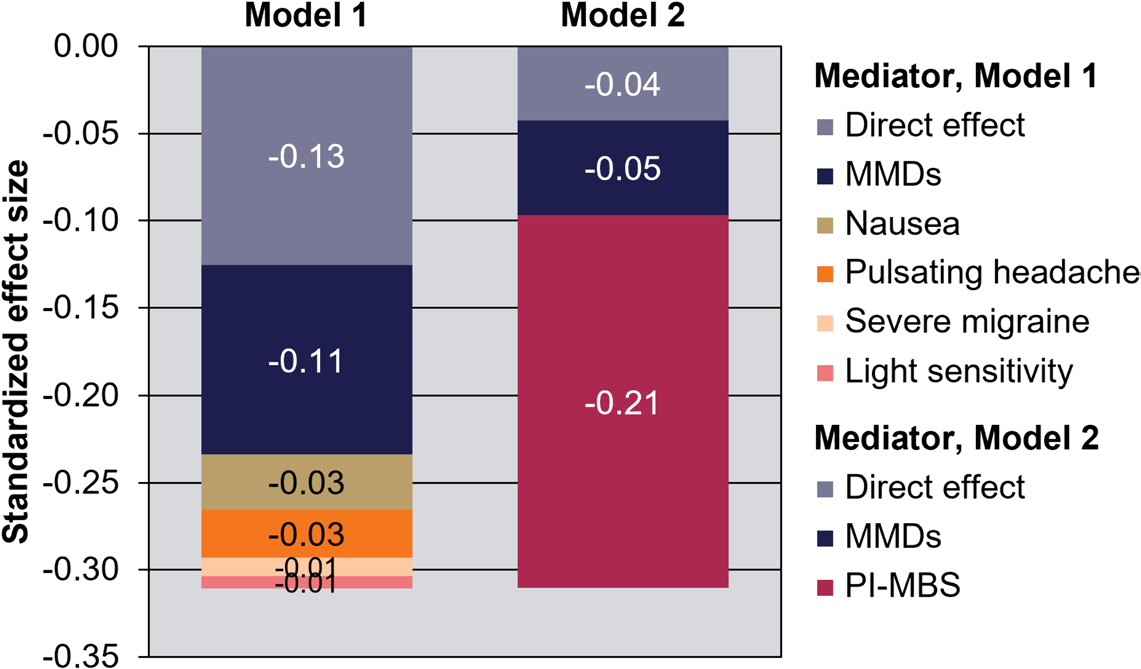
Direct and mediated effect of eptinezumab on HRQoL HRQoL, health-related quality of life; MMDs, monthly migraine days; PI-MBS, patient-identified most bothersome symptom

Using Model 2, which incorporated PI-MBS as a mediator, substantially more (approximately 87%) of the impact of eptinezumab on HRQoL was either explained by MMD reduction or PI-MBS improvement, with PI-MBS improvement (69%) greatly exceeding that of MMD reduction (17%) (Fig. 2). Measures of goodness of model fit (χ^2^/df = 2.5, CFI = 0.9960, RMSEA = 0.043, SRMR = 0.011) indicated that Model 2 had a satisfactory fit. Given that the Akaike information criterion of Model 2 (27914) was lower than that of Model 1 (33289), Model 2 provided better fit to data than Model 1. Analyses were conducted on pooled data for two dosages of eptinezumab (100 mg and 300 mg); results were similar when reproduced separately for each dosage (see Supplemental Tables 7 and 8).

## Discussion

Structural equation modeling analysis—through the development of two complementary models for describing sources of HRQoL improvement in patients treated with eptinezumab—showed that the HRQoL burden in migraine was primarily driven by efficacy measures other than the frequency of migraine days (i.e., MMDs). MMDs and improvement in canonical symptoms insufficiently explained how eptinezumab improved patients’ HRQoL, as demonstrated by the amplitude of the direct effect in Model 1 (i.e., 41% of the eptinezumab effect was not explained by included mediators). In particular, the impact on HRQoL was mainly driven by improvement in the most bothersome migraine-associated symptom that patients individually identified (i.e., PI-MBS), as demonstrated by its 69% share of the total effect in Model 2. Based on Model 2 results, the improvement in HRQoL experienced by patients treated with eptinezumab can almost solely be explained by its effect on both MMDs and PI-MBS.

This work helps to corroborate previous work with structural equation modeling that showed headache chronicity to have prominent direct and indirect effects on HRQoL, underscoring the complexity of disease burden [22]. One revelation of our work is that while migraine has both direct and indirect effects on HRQoL, the indirect effects (PI-MBS, MMDs) had a stronger impact on HRQoL compared to the direct effects of migraine for the patient population of DELIVER. Notably, in this DELIVER population, pain with activity was the symptom that patients with migraine reported as being the most bothersome (i.e., contributing the most to the PI-MBS percentage in Model 2), which differed compared to the canonical symptoms with the greatest effect on HRQoL in Model 1 (nausea, severe migraine, pulsating headache, and light sensitivity). Previous work highlighted how pain severity is a key, yet not as widely recognized, indirect mediator of quality of life for a patient with migraine—negative pain perception and related emotions can significantly reduce quality of life for patients with migraine [23, 24]. Understanding the relative contributions of all mediators, indirect and direct, on HRQoL is especially important for a difficult-to-treat migraine population such as the DELIVER population, if patients are trying to determine the cause of past treatment failures more precisely. This knowledge may also have clinical implications; focusing on the effect on the most bothersome symptom may facilitate the assessment of individual response to treatment in the clinical setting and help guide clinical decision making, especially as newer economic evaluations view migraine as a spectrum disorder [25].

From the PROMISE-2 clinical trial, it is known that eptinezumab treatment results in significantly more patients reporting “much improved” or “very much improved” on their PI-MBS questionnaires versus placebo [13], explaining the strong effect of eptinezumab on HRQoL. Compared to some functional measures of HRQoL, PI-MBS assessments can directly probe specific migraine symptoms and can help clinicians understand the individual impact of each migraine symptom on a patient’s life, beyond the symptoms integral to the diagnostic criteria of migraine such as migraine frequency. This motivated the development of structural equations that included PI-MBS as a potential mediator of effects on HRQoL. Physicians should consider factors other than migraine frequency to have a fuller picture of a preventive treatment’s impact on a patient’s HRQoL. Moving toward a more holistic and patient-customized definition of treatment success can facilitate this. Economic evaluations of migraine therapies should consider treatment effects beyond migraine frequency reduction in order to not underestimate impact of preventive treatment and non-headache migraine-related symptoms on HRQoL and health utilities.

### Limitations

The analysis does not account for the wide range of most bothersome symptoms that can be reported by patients with migraine in clinical studies; up to 23 distinct symptoms were reported at baseline, but only nine options could be chosen at baseline (including “other”). Furthermore, the models discussed here do not account for route of administration, which can vary depending on the migraine treatment. However, while all variables were not included in the final models discussed here, the non-significant paths were removed during screening and the most significant variables to Models 1 and 2 were considered. Additionally, some missing data, such as missing eDiary entries from DELIVER, were not accounted for in this post hoc analysis, given that eDiary compliance was high. At all 4-week intervals, the proportion of patients with ≥14 or ≥ 21 days of compliance was > 96% and > 90%, respectively, for all the treatment groups. The denominators for the summaries of a given variable were based on the number of patients with non-missing values at a given visit or during the assessment period. The relatively short term (24 weeks) of the placebo-controlled portion of DELIVER may not fully capture the long-term effects of improved disease control on HRQoL. In Model 1, the total standardized effect size was 0.312; a reason for the seemingly small effect size could be that we averaged the effects per period, rather than evaluating them over the entire clinical trial. The accumulated effect size over the entire 24-week treatment period would be considerably greater. The overall effect on HRQoL over the entire trial can be found in Goadsby et al. [17]. Finally, the trial population of DELIVER may not be fully representative of the overall migraine population—for example, due to a higher prevalence of co-morbidities or a higher number of prior preventive treatment failures—which in turn may further underestimate the impact of migraine on HRQoL.

## Conclusion

In this exploratory analysis of the DELIVER clinical trial, structural equation modeling was used to identify the impact of eptinezumab on HRQoL through various indirect mediators of treatment efficacy: changes in MMDs, migraine severity, canonical symptoms, and PI-MBS. Comparisons were made to the direct effect of eptinezumab on HRQoL. Eighty-six percent of the impact of eptinezumab on HRQoL was explained by reductions in monthly migraine days and improvements to PI-MBS, with improvements in PI-MBS (69%) contributing more than reductions in MMDs (17%) to the total treatment effect of eptinezumab. To the best of our knowledge, this is the first study to examine the additional sources of HRQoL improvement in patients with migraine, primarily improvements to canonical symptoms and PI-MBS, beyond MMD reduction.

### Supplementary Information

**Supplementary material 1.**

## Data Availability

All data generated or analyzed during this study are included in this published article (and its supplementary content).

## Abbreviations

CFI: Comparative fit index
CGRP: Calcitonin gene-related peptide
EF: Emotional Function
HIT-6: 6-item Headache Impact Test
HRQoL: Health-related quality of life
ICHD-3: International Classification of Headache Disorders, 3rd edition
MMDs: Monthly migraine days
MSQ: Migraine-Specific Quality of Life Questionnaire
PGIC: Patient Global Impression of Change
PI-MBS: Patient-identified most bothersome symptom
RMSEA: Root mean square error of approximation
RP: Role-Preventive
RR: Role-Restrictive
SEM: Structural equation modeling
SRMR: Standardized root mean square residual
