# Supplementary material for: Structural equation modeling for identifying the drivers of health-related quality of life improvement experienced by patients with migraine receiving eptinezumab
Source: J Headache Pain. 2024 Mar 28;25(1):45. doi: 10.1186/s10194-024-01752-z (PMC10976712; doi:10.1186/s10194-024-01752-z)
Supplement: Supplementary file 1 — Supplementary material 1. [file 10194_2024_1752_MOESM1_ESM.pdf]

Online Supplementary Material

Supplemental Table 1. Backward elimination method analysis for selecting a subset of explanatory variables for the structural equation modeling of MSQ-derived mediators of HRQoL.

Supplemental Table 2. Model fit statistics.

Supplemental Table 3. Model regression coefficients for HRQoL predicted by effect modifiers.

Supplemental Table 4. Model regression coefficients for mediated treatment effects.

Supplemental Table 5. Summary of models

Supplemental Table 6. Comparison of fit statistics across all models

Supplemental Model Details

- **Model A**
- **Model B**
- **Model C**
- **Model D**
- **Model E**
- **Model F**
- **Model G**
- **Model 1**
- **Model 2**

Supplemental Table 7. Estimated coefficients of Final Model 1 separately by dosage of eptinezumab (100 mg vs 300 mg)

Supplemental Table 8. Mediation analysis with Final Model 1 separately by dosage of eptinezumab (100 mg vs 300 mg)

Supplemental Table 1. Backward elimination method analysis for selecting a subset of explanatory variables for the structural equation modeling of MSQ-derived mediators of HRQoL

	Estimate						P-value					
**Model**	**Step a**	**Step b**	**Step c**	**Step d**	**Step e**	**Step f**	**Step a**	**Step b**	**Step c**	**Step d**	**Step e**	**Step f**
Treatment (direct effect)	–4.98	–4.95	–4.98	–5.06	–5.04	–5.05	<0.001	<0.001	<0.001	<0.001	<0.001	<0.001
Monthly migraine days	–1.43	–1.43	–1.43	–1.44	–1.44	–1.44	<0.001	<0.001	<0.001	<0.001	<0.001	<0.001
Severe migraine	–0.10	–0.10	–0.10	–0.10	–0.11	–0.11	<0.001	<0.001	<0.001	<0.001	<0.001	<0.001
Nausea	–8.90	–8.77	–8.80	–9.08	–9.52	–9.66	<0.001	<0.001	<0.001	<0.001	<0.001	<0.001
Sensitivity to light	–3.41	–3.32	–4.56	–4.67	–4.74	–5.54	0.284	0.297	0.084	0.077	0.072	0.033
Pulsating/throbbing	–4.72	–4.61	–4.94	–5.16	–5.13	–5.84	0.102	0.109	0.082	0.068	0.069	0.037
Aggravation by physical activity	–3.27	–3.36	–3.73	–3.86	–4.04	N/A	0.227	0.213	0.159	0.144	0.125	N/A
Vomiting	–4.00	–3.64	–3.52	–3.40	N/A	N/A	0.237	0.277	0.293	0.310	N/A	N/A
One-sidedness	–2.10	–2.07	–2.00	N/A	N/A	N/A	0.462	0.469	0.484	N/A	N/A	N/A
Sensitivity to sound	–2.36	–2.25	N/A	N/A	N/A	N/A	0.461	0.483	N/A	N/A	N/A	N/A
Aura	1.91	N/A	N/A	N/A	N/A	N/A	0.468	N/A	N/A	N/A	N/A	N/A

The direct effect is the amount of eptinezumab effect not otherwise explained by the efficacy mediators explored. Other variables were captured through the electronic headache diary, which recorded the start/stop date/times of experienced headache episodes and recorded headache characteristics used for classifying headache episodes as migraine attacks. Severe migraine was calculated as the percentage of migraine attacks that were rated as severe. Nausea, sensitivity to light, pulsating/throbbing, aggravation by physical activity, vomiting, one-sidedness, sensitivity to sound, and aura represent the percentage of migraine attacks with the respective symptom present.

HRQoL, health-related quality of life; MSQ, Migraine-Specific Quality of Life Questionnaire; N/A, not applicable (removed from model).

Supplemental Table 2. Model fit statistics

**Model**	**N observations**	**N parameters**	**Chi-square**	**Degrees of freedom**	**P-value**	**Comparative fit index**	**Akaike information criterion**	**Root mean square error of approximation**	**Standardized root mean square residual**
1	836	32	17	12	0.140	0.998	33289	0.023	0.009
2	836	14	15	6	0.0180	0.996	27914	0.043	0.011

Supplemental Table 3. Model regression coefficients for HRQoL predicted by effect modifiers

**Model**	**Variable**	**Estimate**	**Standard error**	**P-value**
Model 1	Monthly migraine days	–1.443	0.135	<0.001
	Severe migraine	–0.115	0.026	<0.001
	Nausea	–9.660	2.403	<0.001
	Sensitivity to light	–5.537	2.591	0.033
	Pulsating/throbbing	–5.839	2.795	0.037
	Treatment	–5.047	1.288	<0.001
Model 2	Monthly migraine days	–0.722	0.133	<0.001
	Change in PI-MBS	–9.247	0.647	<0.001
	Treatment	–1.703	1.220	0.163

HRQoL, health-related quality of life; PI-MBS, patient-identified most bothersome symptom.

Supplemental Table 4. Model regression coefficients for mediated treatment effects

**Model**	**Variable**	**Estimate**	**Standard error**	**P-value**
Model 1	Monthly migraine days	3.021	0.331	<0.001
	Severe migraine	11.146	1.832	<0.001
	Nausea	0.114	0.020	<0.001
	Sensitivity to light	0.078	0.018	<0.001
	Pulsating/throbbing	0.049	0.016	0.002
Model 2	Monthly migraine days	3.021	0.331	<0.001
	Change in PI-MBS	0.926	0.074	<0.001

PI-MBS, patient-identified most bothersome symptom.

Supplemental Table 5. Summary of models

		Mediators of treatment response										
**Model**	**Measures of latent variable**	MMDs	Severity	Nausea	Light sensitivity	Pulsating	Physical activity	Vomiting	One-sidedness	Sound sensitivity	Aura	PI-MBS
A	Individual items of the MSQ and HIT-6	x	x	x	x	x	x	x	x	x	x	
B	Individual items of the MSQ	x	x	x	x	x	x	x	x	x	x	
C	Three domains of the MSQ	x	x	x	x	x	x	x	x	x	x	
D	Three domains of the MSQ	x	x	x	x	x	x	x	x	x		
E	Three domains of the MSQ	x	x	x	x	x	x	x	x			
F	Three domains of the MSQ	x	x	x	x	x	x	x				
G	Three domains of the MSQ	x	x	x	x	x	x					
Model 1	Three domains of the MSQ	x	x	x	x	x						x
Model 2	Three domains of the MSQ	x										x

Models A-G are development versions with different definitions of the latent variable and different mediators of treatment response included. Starting with Model C, mediators were eliminated one at a time until only statistically significant mediators (at $p<0.05$) remained in the model. This resulted in Models D-G and Model 1, which is the final model with only significant mediators. Model 2 uses PI-MBS as mediator instead of the canonical symptoms.

HIT-6, 6-item Headache Impact Test; MMDs, monthly migraine days; MSQ, Migraine-Specific Quality of Life Questionnaire; PI-MBS, patient-identified most bothersome symptom.

Supplemental Table 6. Comparison of fit statistics across all models

**Model**	**Number of parameters**	**Degrees of freedom**	**Chi square statistic**	**Comparative Fit Index**	**RMSEA**	**SRMR**	**AIC**
A	116	379	2,473.9	0.867	0.081	0.046	55,787
B	104	220	1,105.6	0.923	0.070	0.033	40,798
C	82	22	38.5	0.995	0.030	0.010	31,455
D	70	20	31.8	0.997	0.027	0.009	31,619
E	59	18	30.9	0.996	0.029	0.010	32,108
F	49	16	27.7	0.996	0.030	0.010	32,407
G	40	14	17.6	0.999	0.018	0.009	32,992
Final Model 1	32	12	16.6	0.998	0.021	0.009	33,171
Final Model 2	14	6	14.6	0.996	0.041	0.011	27,814

AIC, Akaike information criterion; RMSEA, root mean square error of approximation; SRMR, standardized root mean square residual.

Model Details: Model A

Model A includes all individual items of the Migraine-Specific Quality of Life Questionnaire (MSQ) and 6-item Headache Impact Test (HIT-6) scales as measures of health-related quality of life (HRQoL), and monthly migraine days (MMDs) and all canonical symptoms as mediators.

Figure S1.A. Model A structure

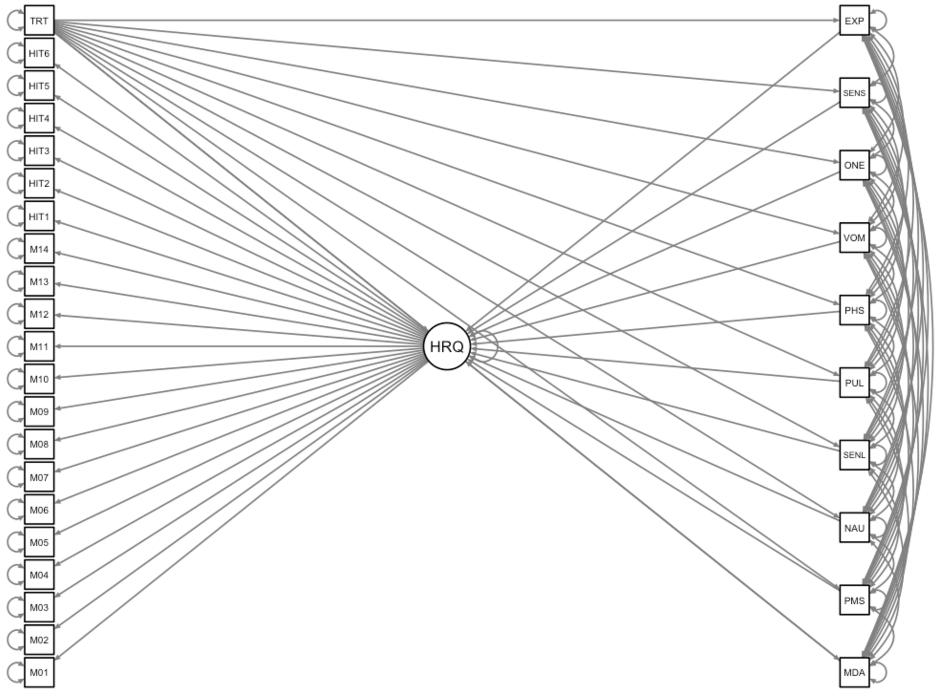

EXP, aura; HIT1–HIT6, 6-item Headache Impact Test items 1–6; HRQ, health-related quality of life; M01–M14, Migraine-Specific Quality of Life Questionnaire items 1–14; MDA, monthly migraine days; NAU, nausea; ONE, one-sidedness; PHS, aggravation by physical activity; PMS, migraine severity; PUL, pulsating/throbbing headache; SENL, sensitivity to light; SENS, sensitivity to sound; TRT, treatment (direct effect); VOM, vomiting.

Table S2.A. Model A fit statistics

Number of parameters	116
Degrees of freedom	379
Chi square statistic	2,473.854
Comparative Fit Index	0.867
RMSEA	0.081
SRMR	0.046
AIC	55,787

AIC, Akaike information criterion; RMSEA, root mean square error of approximation; SRMR, standardized root mean square residual.

Table S3.A. Model A latent variable

Left-hand side variable	Right-hand side variable	Estimate	SE	Z-score	p-value	std.all
HRQoL						
	M01	1.000	0.000			0.785
	M02	1.130	0.041	27.606	<0.001	0.836
	M03	1.112	0.038	29.318	<0.001	0.874
	M04	1.125	0.039	28.815	<0.001	0.863
	M05	1.105	0.039	28.607	<0.001	0.859
	M06	1.078	0.039	27.619	<0.001	0.837
	M07	0.996	0.039	25.300	<0.001	0.783
	M08	0.896	0.039	23.248	<0.001	0.732
	M09	0.918	0.041	22.330	<0.001	0.709
	M10	0.968	0.040	24.400	<0.001	0.761
	M11	1.076	0.047	22.669	<0.001	0.718
	M12	1.104	0.046	24.160	<0.001	0.755
	M13	0.917	0.047	19.654	<0.001	0.638
	M14	0.848	0.046	18.505	<0.001	0.606
	HIT1	-0.770	0.041	-18.762	<0.001	-0.613
	HIT2	-0.934	0.046	-20.428	<0.001	-0.659
	HIT3	-0.880	0.050	-17.753	<0.001	-0.584
	HIT4	-1.180	0.046	-25.878	<0.001	-0.797
	HIT5	-1.299	0.055	-23.818	<0.001	-0.747
	HIT6	-1.240	0.050	-24.830	<0.001	-0.772

HIT1–HIT6, 6-item Headache Impact Test items 1–6; HRQoL, health-related quality of life; M01–M14, Migraine-Specific Quality of Life Questionnaire items 1–14; SE, standard error.

Table S4.A. Model A regressions

Left-hand side variable	Right-hand side variable	Estimate	SE	Z-score	p-value	std.all
HRQoL	MMDs	-0.075	0.007	-11.456	<0.001	-0.367
HRQoL	Severe migraine	-0.005	0.001	-4.121	<0.001	-0.140
HRQoL	Nausea	-0.374	0.119	-3.135	0.002	-0.108
HRQoL	Light sensitivity	-0.255	0.153	-1.664	0.096	-0.065
HRQoL	Pulsating/throbbing	-0.181	0.139	-1.299	0.194	-0.040
HRQoL	Aggravation by phys. act.	-0.188	0.131	-1.435	0.151	-0.046
HRQoL	Vomiting	-0.136	0.165	-0.825	0.409	-0.026
HRQoL	One-sidedness	-0.156	0.138	-1.130	0.258	-0.034
HRQoL	Sound sensitivity	-0.166	0.155	-1.073	0.283	-0.042
HRQoL	Aura	0.050	0.127	0.397	0.692	0.012
MMDs	Treatment (direct effect)	-3.030	0.332	-9.116	<0.001	-0.301
Severe migraine	Treatment (direct effect)	-11.117	1.826	-6.089	<0.001	-0.206
Nausea	Treatment (direct effect)	-0.114	0.020	-5.643	<0.001	-0.192
Light sensitivity	Treatment (direct effect)	-0.078	0.018	-4.378	<0.001	-0.150
Pulsating/throbbing	Treatment (direct effect)	-0.049	0.016	-3.081	0.002	-0.106
Aggravation by phys. act.	Treatment (direct effect)	-0.050	0.017	-2.874	0.004	-0.099
Vomiting	Treatment (direct effect)	-0.032	0.013	-2.352	0.019	-0.081
One-sidedness	Treatment (direct effect)	-0.065	0.015	-4.271	<0.001	-0.146
Sound sensitivity	Treatment (direct effect)	-0.084	0.018	-4.707	<0.001	-0.161
Aura	Treatment (direct effect)	-0.042	0.016	-2.591	0.010	-0.089
HRQoL	Treatment (direct effect)	0.287	0.062	4.594	<0.001	0.139

HRQoL, health-related quality of life; MMDs, monthly migraine days; Phys. act., physical activity; SE, standard error.

Table S5.A. Model A covariances

Left-hand side variable	Right-hand side variable	Estimate	SE	Z-score	p-value	std.all
MMDs	Severe migraine	23.139	4.036	5.734	<0.001	0.203
MMDs	Nausea	0.146	0.044	3.321	<0.001	0.116
Severe migraine	Nausea	2.770	0.259	10.712	<0.001	0.400
MMDs	Light sensitivity	0.138	0.039	3.540	<0.001	0.124
Severe migraine	Light sensitivity	1.666	0.220	7.574	<0.001	0.272
Nausea	Light sensitivity	0.025	0.003	10.064	<0.001	0.372
MMDs	Pulsating/throbbing	0.112	0.034	3.242	0.001	0.113
Severe migraine	Pulsating/throbbing	0.986	0.191	5.157	<0.001	0.182
Nausea	Pulsating/throbbing	0.016	0.002	7.295	<0.001	0.261
Light sensitivity	Pulsating/throbbing	0.014	0.002	7.408	<0.001	0.266
MMDs	Aggravation by phys. act.	0.118	0.038	3.116	0.002	0.109
Severe migraine	Aggravation by phys. act.	1.802	0.216	8.360	<0.001	0.303
Nausea	Aggravation by phys. act.	0.016	0.002	6.681	<0.001	0.238
Light sensitivity	Aggravation by phys. act.	0.018	0.002	8.739	<0.001	0.318
Pulsating/throbbing	Aggravation by phys. act.	0.014	0.002	7.322	<0.001	0.262
MMDs	Vomiting	0.065	0.029	2.206	0.027	0.077
Severe migraine	Vomiting	1.652	0.170	9.693	<0.001	0.357
Nausea	Vomiting	0.016	0.002	8.816	<0.001	0.321
Light sensitivity	Vomiting	0.008	0.002	5.165	<0.001	0.182
Pulsating/throbbing	Vomiting	0.004	0.001	3.186	0.001	0.111
Aggravation by phys. act.	Vomiting	0.008	0.002	5.342	<0.001	0.188
MMDs	One-sidedness	0.089	0.033	2.680	0.007	0.093
Severe migraine	One-sidedness	0.143	0.181	0.792	0.428	0.027
Nausea	One-sidedness	0.012	0.002	5.782	<0.001	0.204
Light sensitivity	One-sidedness	0.008	0.002	4.558	<0.001	0.160
Pulsating/throbbing	One-sidedness	0.008	0.002	5.190	<0.001	0.183
Aggravation by phys. act.	One-sidedness	0.007	0.002	3.931	<0.001	0.137
Vomiting	One-sidedness	0.000	0.001	0.319	0.749	0.011
MMDs	Sound sensitivity	0.185	0.039	4.735	<0.001	0.166
Severe migraine	Sound sensitivity	1.431	0.218	6.575	<0.001	0.234
Nausea	Sound sensitivity	0.019	0.002	7.967	<0.001	0.287
Light sensitivity	Sound sensitivity	0.039	0.002	15.627	<0.001	0.644
Pulsating/throbbing	Sound sensitivity	0.017	0.002	8.870	<0.001	0.323
Aggravation by phys. act.	Sound sensitivity	0.022	0.002	10.086	<0.001	0.373
Vomiting	Sound sensitivity	0.005	0.002	3.342	<0.001	0.117
One-sidedness	Sound sensitivity	0.006	0.002	3.376	<0.001	0.118
MMDs	Aura	0.102	0.036	2.866	0.004	0.100
Severe migraine	Aura	0.485	0.195	2.489	0.013	0.087
Nausea	Aura	0.010	0.002	4.766	<0.001	0.167
Light sensitivity	Aura	0.008	0.002	4.409	<0.001	0.155
Pulsating/throbbing	Aura	0.006	0.002	3.412	<0.001	0.119
Aggravation by phys. act.	Aura	0.003	0.002	1.392	0.164	0.048
Vomiting	Aura	0.007	0.001	5.066	<0.001	0.178
One-sidedness	Aura	0.003	0.002	1.761	0.078	0.061
Sound sensitivity	Aura	0.008	0.002	4.155	<0.001	0.145

MMDs, monthly migraine days; Phys. act., physical activity; SE, standard error.

Table S6.A. Model A variances

Left-hand side variable	Estimate	SE	Z-score	p-value	std.all
M01	0.599	0.031	19.352	<0.001	0.385
M02	0.525	0.028	18.870	<0.001	0.300
M03	0.366	0.020	18.270	<0.001	0.236
M04	0.415	0.022	18.477	<0.001	0.255
M05	0.417	0.022	18.554	<0.001	0.263
M06	0.477	0.025	18.866	<0.001	0.300
M07	0.600	0.031	19.363	<0.001	0.387
M08	0.665	0.034	19.644	<0.001	0.463
M09	0.800	0.041	19.741	<0.001	0.497
M10	0.652	0.033	19.499	<0.001	0.421
M11	1.043	0.053	19.707	<0.001	0.485
M12	0.879	0.045	19.532	<0.001	0.430
M13	1.177	0.059	19.957	<0.001	0.593
M14	1.190	0.059	20.027	<0.001	0.633
HIT1	0.945	0.047	20.012	<0.001	0.625
HIT2	1.091	0.055	19.903	<0.001	0.566
HIT3	1.432	0.071	20.067	<0.001	0.659
HIT4	0.768	0.040	19.261	<0.001	0.365
HIT5	1.282	0.065	19.576	<0.001	0.442
HIT6	1.001	0.052	19.437	<0.001	0.405
MMDs	20.783	1.018	20.408	<0.001	0.909
Severe migraine	626.976	30.722	20.408	<0.001	0.957
Nausea	0.077	0.004	20.408	<0.001	0.963
Light sensitivity	0.060	0.003	20.408	<0.001	0.978
Pulsating/throbbing	0.047	0.002	20.408	<0.001	0.989
Aggravation by phys. act.	0.057	0.003	20.408	<0.001	0.990
Vomiting	0.034	0.002	20.408	<0.001	0.993
One-sidedness	0.044	0.002	20.408	<0.001	0.979
Sound sensitivity	0.060	0.003	20.408	<0.001	0.974
Aura	0.050	0.002	20.408	<0.001	0.992
HRQoL	0.598	0.045	13.356	<0.001	0.624
Treatment (direct effect)	0.226	0.000			1.000

HIT1–HIT6, 6-item Headache Impact Test items 1–6; HRQoL, health-related quality of life; M01–M14, Migraine-Specific Quality of Life Questionnaire items 1–14; MMDs, monthly migraine days; Phys. act., physical activity; SE, standard error.

Model Details: Model B

Model B includes all individual items of the Migraine-Specific Quality of Life Questionnaire (MSQ) as measures of health-related quality of life (HRQoL), and monthly migraine days (MMDs) and all canonical symptoms as mediators.

Figure S1.B. Model B structure

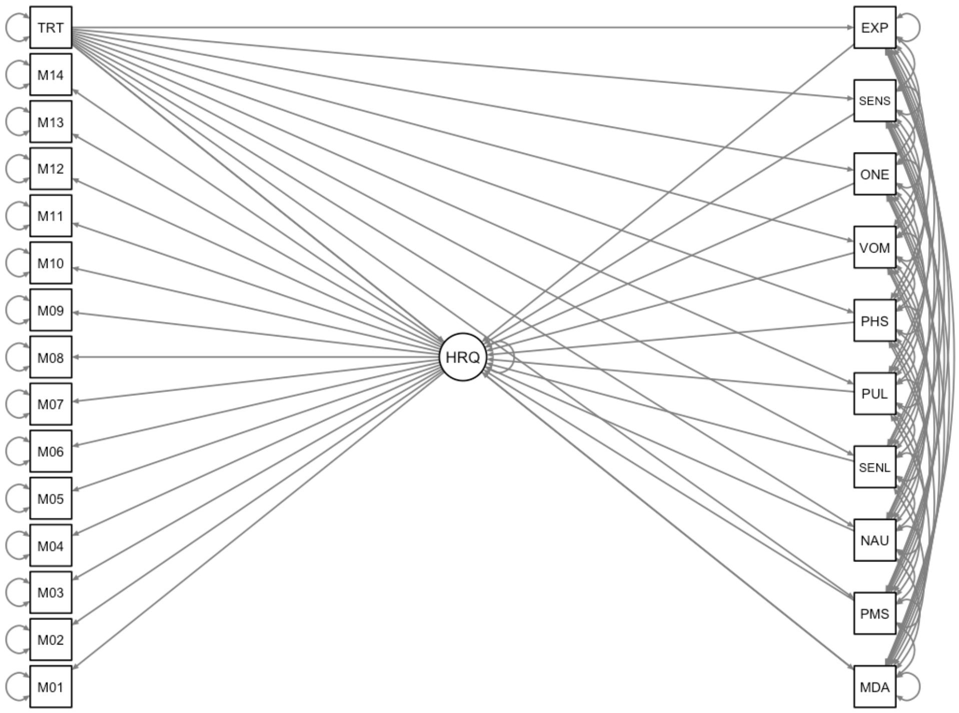

EXP, aura; HRQ, health-related quality of life; M01–M14, Migraine-Specific Quality of Life Questionnaire items 1–14; MDA, monthly migraine days; NAU, nausea; ONE, one-sidedness; PHS, aggravation by physical activity; PMS, migraine severity; PUL, pulsating/throbbing headache; SENL, sensitivity to light; SENS, sensitivity to sound; TRT, treatment (direct effect); VOM, vomiting.

Table S2.B. Model B fit statistics

Number of parameters	104
Degrees of freedom	220
Chi square statistic	1,105.581
Comparative Fit Index	0.923
RMSEA	0.070
SRMR	0.033
AIC	40,798

AIC, Akaike information criterion; RMSEA, root mean square error of approximation; SRMR, standardized root mean square residual.

Table S3.B. Model B latent variable

Left-hand side variable	Right-hand side variable	Estimate	SE	Z-score	p-value	std.all
HRQoL						
	M01	1.000	0.000			0.783
	M02	1.130	0.041	27.393	<0.001	0.836
	M03	1.121	0.038	29.367	<0.001	0.880
	M04	1.142	0.039	29.155	<0.001	0.875
	M05	1.117	0.039	28.769	<0.001	0.866
	M06	1.094	0.039	27.909	<0.001	0.847
	M07	0.999	0.040	25.194	<0.001	0.784
	M08	0.917	0.039	23.731	<0.001	0.748
	M09	0.935	0.041	22.653	<0.001	0.720
	M10	0.983	0.040	24.675	<0.001	0.771
	M11	1.098	0.048	23.087	<0.001	0.732
	M12	1.101	0.046	23.899	<0.001	0.752
	M13	0.946	0.047	20.250	<0.001	0.657
	M14	0.867	0.046	18.862	<0.001	0.618

HRQoL, health-related quality of life; M01–M14, Migraine-Specific Quality of Life Questionnaire items 1–14; SE, standard error.

Table S4.B. Model B regressions

Left-hand side variable	Right-hand side variable	Estimate	SE	Z-score	p-value	std.all
HRQoL	MMDs	-0.070	0.007	-10.461	<0.001	-0.345
HRQoL	Severe migraine	-0.005	0.001	-3.741	<0.001	-0.132
HRQoL	Nausea	-0.410	0.123	-3.321	<0.001	-0.118
HRQoL	Light sensitivity	-0.197	0.159	-1.238	0.216	-0.050
HRQoL	Pulsating/throbbing	-0.235	0.144	-1.634	0.102	-0.052
HRQoL	Aggravation by phys. act.	-0.173	0.135	-1.278	0.201	-0.042
HRQoL	Vomiting	-0.177	0.171	-1.037	0.300	-0.034
HRQoL	One-sidedness	-0.117	0.142	-0.819	0.413	-0.025
HRQoL	Sound sensitivity	-0.122	0.160	-0.761	0.447	-0.031
HRQoL	Aura	0.064	0.132	0.487	0.626	0.015
MMDs	Treatment (direct effect)	-3.030	0.332	-9.116	<0.001	-0.301
Severe migraine	Treatment (direct effect)	-11.117	1.826	-6.089	<0.001	-0.206
Nausea	Treatment (direct effect)	-0.114	0.020	-5.643	<0.001	-0.192
Light sensitivity	Treatment (direct effect)	-0.078	0.018	-4.378	<0.001	-0.150
Pulsating/throbbing	Treatment (direct effect)	-0.049	0.016	-3.081	0.002	-0.106
Aggravation by phys. act.	Treatment (direct effect)	-0.050	0.017	-2.874	0.004	-0.099
Vomiting	Treatment (direct effect)	-0.032	0.013	-2.352	0.019	-0.081
One-sidedness	Treatment (direct effect)	-0.065	0.015	-4.271	<0.001	-0.146
Sound sensitivity	Treatment (direct effect)	-0.084	0.018	-4.707	<0.001	-0.161
Aura	Treatment (direct effect)	-0.042	0.016	-2.591	0.010	-0.089
HRQoL	Treatment (direct effect)	0.253	0.065	3.923	<0.001	0.123

HRQoL, health-related quality of life; MMDs, monthly migraine days; Phys. act., physical activity; SE, standard error.

Table S5.B. Model B covariances

Left-hand side variable	Right-hand side variable	Estimate	SE	Z-score	p-value	std.all
MMDs	Severe migraine	23.139	4.036	5.734	<0.001	0.203
MMDs	Nausea	0.146	0.044	3.321	<0.001	0.116
Severe migraine	Nausea	2.770	0.259	10.712	<0.001	0.400
MMDs	Light sensitivity	0.138	0.039	3.540	<0.001	0.124
Severe migraine	Light sensitivity	1.666	0.220	7.574	<0.001	0.272
Nausea	Light sensitivity	0.025	0.003	10.064	<0.001	0.372
MMDs	Pulsating/throbbing	0.112	0.034	3.242	0.001	0.113
Severe migraine	Pulsating/throbbing	0.986	0.191	5.157	<0.001	0.182
Nausea	Pulsating/throbbing	0.016	0.002	7.295	<0.001	0.261
Light sensitivity	Pulsating/throbbing	0.014	0.002	7.408	<0.001	0.266
MMDs	Aggravation by phys. act.	0.118	0.038	3.116	0.002	0.109
Severe migraine	Aggravation by phys. act.	1.802	0.216	8.360	<0.001	0.303
Nausea	Aggravation by phys. act.	0.016	0.002	6.681	<0.001	0.238
Light sensitivity	Aggravation by phys. act.	0.018	0.002	8.739	<0.001	0.318
Pulsating/throbbing	Aggravation by phys. act.	0.014	0.002	7.322	<0.001	0.262
MMDs	Vomiting	0.065	0.029	2.206	0.027	0.077
Severe migraine	Vomiting	1.652	0.170	9.693	<0.001	0.357
Nausea	Vomiting	0.016	0.002	8.816	<0.001	0.321
Light sensitivity	Vomiting	0.008	0.002	5.165	<0.001	0.182
Pulsating/throbbing	Vomiting	0.004	0.001	3.186	0.001	0.111
Aggravation by phys. act.	Vomiting	0.008	0.002	5.342	<0.001	0.188
MMDs	One-sidedness	0.089	0.033	2.680	0.007	0.093
Severe migraine	One-sidedness	0.143	0.181	0.792	0.428	0.027
Nausea	One-sidedness	0.012	0.002	5.781	<0.001	0.204
Light sensitivity	One-sidedness	0.008	0.002	4.558	<0.001	0.160
Pulsating/throbbing	One-sidedness	0.008	0.002	5.190	<0.001	0.183
Aggravation by phys. act.	One-sidedness	0.007	0.002	3.931	<0.001	0.137
Vomiting	One-sidedness	0.000	0.001	0.319	0.749	0.011
MMDs	Sound sensitivity	0.185	0.039	4.735	<0.001	0.166
Severe migraine	Sound sensitivity	1.431	0.218	6.575	<0.001	0.234
Nausea	Sound sensitivity	0.019	0.002	7.967	<0.001	0.287
Light sensitivity	Sound sensitivity	0.039	0.002	15.627	<0.001	0.644
Pulsating/throbbing	Sound sensitivity	0.017	0.002	8.870	<0.001	0.323
Aggravation by phys. act.	Sound sensitivity	0.022	0.002	10.086	<0.001	0.373
Vomiting	Sound sensitivity	0.005	0.002	3.342	<0.001	0.117
One-sidedness	Sound sensitivity	0.006	0.002	3.376	<0.001	0.118
MMDs	Aura	0.102	0.036	2.866	0.004	0.100
Severe migraine	Aura	0.485	0.195	2.489	0.013	0.087
Nausea	Aura	0.010	0.002	4.766	<0.001	0.167
Light sensitivity	Aura	0.008	0.002	4.409	<0.001	0.155
Pulsating/throbbing	Aura	0.006	0.002	3.412	<0.001	0.119
Aggravation by phys. act.	Aura	0.003	0.002	1.392	0.164	0.048
Vomiting	Aura	0.007	0.001	5.066	<0.001	0.178
One-sidedness	Aura	0.003	0.002	1.761	0.078	0.061
Sound sensitivity	Aura	0.008	0.002	4.155	<0.001	0.145

MMDs, monthly migraine days; Phys. act., physical activity; SE, standard error.

Table S6.B. Model B variances

Left-hand side variable	Estimate	SE	Z-score	p-value	std.all
M01	0.602	0.031	19.153	<0.001	0.387
M02	0.527	0.028	18.575	<0.001	0.302
M03	0.351	0.020	17.699	<0.001	0.226
M04	0.382	0.021	17.820	<0.001	0.235
M05	0.395	0.022	18.020	<0.001	0.249
M06	0.449	0.024	18.391	<0.001	0.282
M07	0.597	0.031	19.147	<0.001	0.385
M08	0.632	0.033	19.405	<0.001	0.441
M09	0.773	0.040	19.555	<0.001	0.481
M10	0.628	0.033	19.247	<0.001	0.405
M11	1.000	0.051	19.498	<0.001	0.465
M12	0.889	0.046	19.379	<0.001	0.434
M13	1.128	0.057	19.810	<0.001	0.569
M14	1.161	0.058	19.920	<0.001	0.618
MMDs	20.783	1.018	20.408	<0.001	0.909
Severe migraine	626.976	30.722	20.408	<0.001	0.957
Nausea	0.077	0.004	20.408	<0.001	0.963
Light sensitivity	0.060	0.003	20.408	<0.001	0.978
Pulsating/throbbing	0.047	0.002	20.408	<0.001	0.989
Aggravation by phys. act.	0.057	0.003	20.408	<0.001	0.990
Vomiting	0.034	0.002	20.408	<0.001	0.993
One-sidedness	0.044	0.002	20.408	<0.001	0.979
Sound sensitivity	0.060	0.003	20.408	<0.001	0.974
Aura	0.050	0.002	20.408	<0.001	0.992
HRQoL	0.636	0.048	13.298	<0.001	0.666
Treatment (direct effect)	0.226	0.000			1.000

HRQoL, health-related quality of life; M01–M14, Migraine-Specific Quality of Life Questionnaire items 1–14; MMDs, monthly migraine days; Phys. act., physical activity; SE, standard error.

Model Details: Model C

Model C includes the three domains of the Migraine-Specific Quality of Life Questionnaire (MSQ) as measures of health-related quality of life (HRQoL), and monthly migraine days (MMDs) and all canonical symptoms as mediators.

Figure S1.C. Model C structure

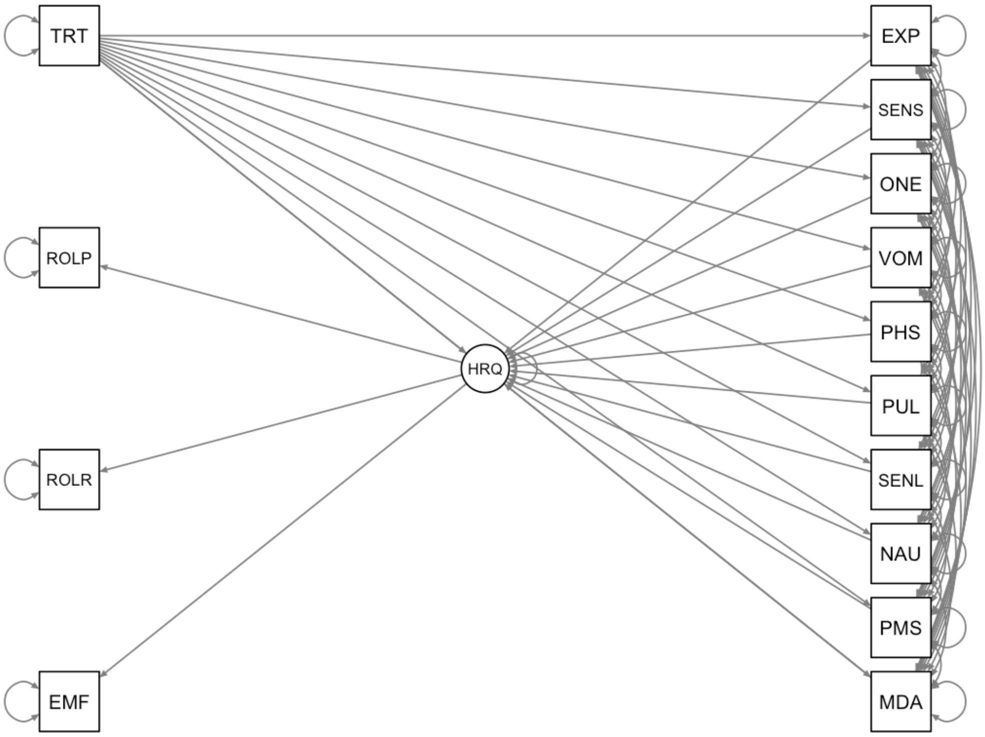

EMF, MSQ Emotional Function; EXP, aura; HRQ, health-related quality of life; MDA, monthly migraine days; NAU, nausea; ONE, one-sidedness; PHS, aggravation by physical activity; PMS, migraine severity; PUL, pulsating/throbbing headache; ROLP, MSQ Role Function-Preventive; ROLR, MSQ Role Function-Restrictive; SENL, sensitivity to light; SENS, sensitivity to sound; TRT, treatment (direct effect); VOM, vomiting.

Table S2.C. Model C fit statistics

Number of parameters	82
Degrees of freedom	22
Chi square statistic	38.470
Comparative Fit Index	0.995
RMSEA	0.030
SRMR	0.010
AIC	31,455

AIC, Akaike information criterion; RMSEA, root mean square error of approximation; SRMR, standardized root mean square residual.

Table S3.C. Model C latent variable

Left-hand side variable	Right-hand side variable	Estimate	SE	Z-score	p-value	std.all
HRQoL						
	MSQ-EF	1.000	0.000			0.784
	MSQ-RR	1.055	0.037	28.887	<0.001	0.920
	MSQ-RP	1.012	0.036	27.919	<0.001	0.879

HRQoL, health-related quality of life; MSQ, Migraine-Specific Quality of Life Questionnaire; MSQ-EF, MSQ Emotional Function; MSQ-RP, MSQ Role Function-Preventive; MSQ-RR, MSQ Role Function-Restrictive; SE, standard error.

Table S4.C. Model C regressions

Left-hand side variable	Right-hand side variable	Estimate	SE	Z-score	p-value	std.all
HRQoL	MMDs	-1.435	0.136	-10.580	<0.001	-0.358
HRQoL	Severe migraine	-0.098	0.027	-3.632	<0.001	-0.131
HRQoL	Nausea	-8.863	2.483	-3.569	<0.001	-0.130
HRQoL	Light sensitivity	-3.404	3.191	-1.067	0.286	-0.044
HRQoL	Pulsating/throbbing	-4.711	2.893	-1.629	0.103	-0.054
HRQoL	Aggravation by phys. act.	-3.496	2.722	-1.284	0.199	-0.044
HRQoL	Vomiting	-4.724	3.433	-1.376	0.169	-0.046
HRQoL	One-sidedness	-2.083	2.864	-0.727	0.467	-0.023
HRQoL	Sound sensitivity	-2.346	3.214	-0.730	0.465	-0.030
HRQoL	Aura	1.797	2.645	0.679	0.497	0.021
MMDs	Treatment (direct effect)	-3.030	0.332	-9.116	<0.001	-0.301
Severe migraine	Treatment (direct effect)	-11.117	1.826	-6.089	<0.001	-0.206
Nausea	Treatment (direct effect)	-0.114	0.020	-5.643	<0.001	-0.192
Light sensitivity	Treatment (direct effect)	-0.078	0.018	-4.378	<0.001	-0.150
Pulsating/throbbing	Treatment (direct effect)	-0.049	0.016	-3.081	0.002	-0.106
Aggravation by phys. act.	Treatment (direct effect)	-0.050	0.017	-2.874	0.004	-0.099
Vomiting	Treatment (direct effect)	-0.032	0.013	-2.351	0.019	-0.081
One-sidedness	Treatment (direct effect)	-0.065	0.015	-4.271	<0.001	-0.146
Sound sensitivity	Treatment (direct effect)	-0.084	0.018	-4.707	<0.001	-0.161
Aura	Treatment (direct effect)	-0.042	0.016	-2.591	0.010	-0.089
HRQoL	Treatment (direct effect)	5.064	1.297	3.905	<0.001	0.125

HRQoL, health-related quality of life; MMDs, monthly migraine days; Phys. act., physical activity; SE, standard error.

Table S5.C. Model C covariances

Left-hand side variable	Right-hand side variable	Estimate	SE	Z-score	p-value	std.all
MMDs	Severe migraine	23.140	4.036	5.734	<0.001	0.203
MMDs	Nausea	0.146	0.044	3.321	<0.001	0.116
Severe migraine	Nausea	2.770	0.259	10.712	<0.001	0.400
MMDs	Light sensitivity	0.138	0.039	3.540	<0.001	0.124
Severe migraine	Light sensitivity	1.666	0.220	7.574	<0.001	0.272
Nausea	Light sensitivity	0.025	0.003	10.064	<0.001	0.372
MMDs	Pulsating/throbbing	0.112	0.034	3.242	0.001	0.113
Severe migraine	Pulsating/throbbing	0.986	0.191	5.157	<0.001	0.182
Nausea	Pulsating/throbbing	0.016	0.002	7.295	<0.001	0.261
Light sensitivity	Pulsating/throbbing	0.014	0.002	7.408	<0.001	0.266
MMDs	Aggravation by phys. act.	0.118	0.038	3.116	0.002	0.109
Severe migraine	Aggravation by phys. act.	1.802	0.216	8.360	<0.001	0.303
Nausea	Aggravation by phys. act.	0.016	0.002	6.681	<0.001	0.238
Light sensitivity	Aggravation by phys. act.	0.018	0.002	8.739	<0.001	0.318
Pulsating/throbbing	Aggravation by phys. act.	0.014	0.002	7.322	<0.001	0.262
MMDs	Vomiting	0.065	0.029	2.206	0.027	0.077
Severe migraine	Vomiting	1.652	0.170	9.693	<0.001	0.357
Nausea	Vomiting	0.016	0.002	8.816	<0.001	0.321
Light sensitivity	Vomiting	0.008	0.002	5.165	<0.001	0.182
Pulsating/throbbing	Vomiting	0.004	0.001	3.186	0.001	0.111
Aggravation by phys. act.	Vomiting	0.008	0.002	5.342	<0.001	0.188
MMDs	One-sidedness	0.089	0.033	2.680	0.007	0.093
Severe migraine	One-sidedness	0.143	0.181	0.792	0.428	0.027
Nausea	One-sidedness	0.012	0.002	5.782	<0.001	0.204
Light sensitivity	One-sidedness	0.008	0.002	4.558	<0.001	0.160
Pulsating/throbbing	One-sidedness	0.008	0.002	5.190	<0.001	0.183
Aggravation by phys. act.	One-sidedness	0.007	0.002	3.931	<0.001	0.137
Vomiting	One-sidedness	0.000	0.001	0.319	0.749	0.011
MMDs	Sound sensitivity	0.185	0.039	4.735	<0.001	0.166
Severe migraine	Sound sensitivity	1.431	0.218	6.575	<0.001	0.234
Nausea	Sound sensitivity	0.019	0.002	7.967	<0.001	0.287
Light sensitivity	Sound sensitivity	0.039	0.002	15.627	<0.001	0.644
Pulsating/throbbing	Sound sensitivity	0.017	0.002	8.870	<0.001	0.323
Aggravation by phys. act.	Sound sensitivity	0.022	0.002	10.086	<0.001	0.373
Vomiting	Sound sensitivity	0.005	0.002	3.342	<0.001	0.117
One-sidedness	Sound sensitivity	0.006	0.002	3.376	<0.001	0.118
MMDs	Aura	0.102	0.036	2.866	0.004	0.100
Severe migraine	Aura	0.485	0.195	2.489	0.013	0.087
Nausea	Aura	0.010	0.002	4.766	<0.001	0.167
Light sensitivity	Aura	0.008	0.002	4.409	<0.001	0.155
Pulsating/throbbing	Aura	0.006	0.002	3.412	<0.001	0.119
Aggravation by phys. act.	Aura	0.003	0.002	1.392	0.164	0.048
Vomiting	Aura	0.007	0.001	5.066	<0.001	0.178
One-sidedness	Aura	0.003	0.002	1.761	0.078	0.061
Sound sensitivity	Aura	0.008	0.002	4.155	<0.001	0.145

MMDs, monthly migraine days; Phys. act., physical activity; SE, standard error.

Table S6.C. Model C variances

Left-hand side variable	Estimate	SE	Z-score	p-value	std.all
MSQ-EF	230.502	13.387	17.219	<0.001	0.385
MSQ-RR	73.956	8.183	9.038	<0.001	0.153
MSQ-RP	111.098	8.743	12.706	<0.001	0.228
MMDs	20.783	1.018	20.408	<0.001	0.909
Severe migraine	626.976	30.722	20.408	<0.001	0.957
Nausea	0.077	0.004	20.408	<0.001	0.963
Light sensitivity	0.060	0.003	20.408	<0.001	0.978
Pulsating/throbbing	0.047	0.002	20.408	<0.001	0.989
Aggravation by phys. act.	0.057	0.003	20.408	<0.001	0.990
Vomiting	0.034	0.002	20.408	<0.001	0.993
One-sidedness	0.044	0.002	20.408	<0.001	0.979
Sound sensitivity	0.060	0.003	20.408	<0.001	0.974
Aura	0.050	0.002	20.408	<0.001	0.992
HRQoL	237.397	18.470	12.853	<0.001	0.645
Treatment (direct effect)	0.226	0.000			1.000

HRQoL, health-related quality of life; MMDs, monthly migraine days; MSQ, Migraine-Specific Quality of Life Questionnaire; MSQ-EF, MSQ Emotional Function; MSQ-RP, MSQ Role Function-Preventive; MSQ-RR, MSQ Role Function-Restrictive; Phys. act., physical activity; SE, standard error.

Model Details: Model D

Model D includes the three domains of the Migraine-Specific Quality of Life Questionnaire (MSQ) as measures of health-related quality of life (HRQoL), and monthly migraine days (MMDs), severity, nausea, sensitivity to light, pulsating/throbbing headache, aggravation by physical activity, vomiting, one-sidedness, and sensitivity to sound as mediators.

Figure S1.D. Model D structure

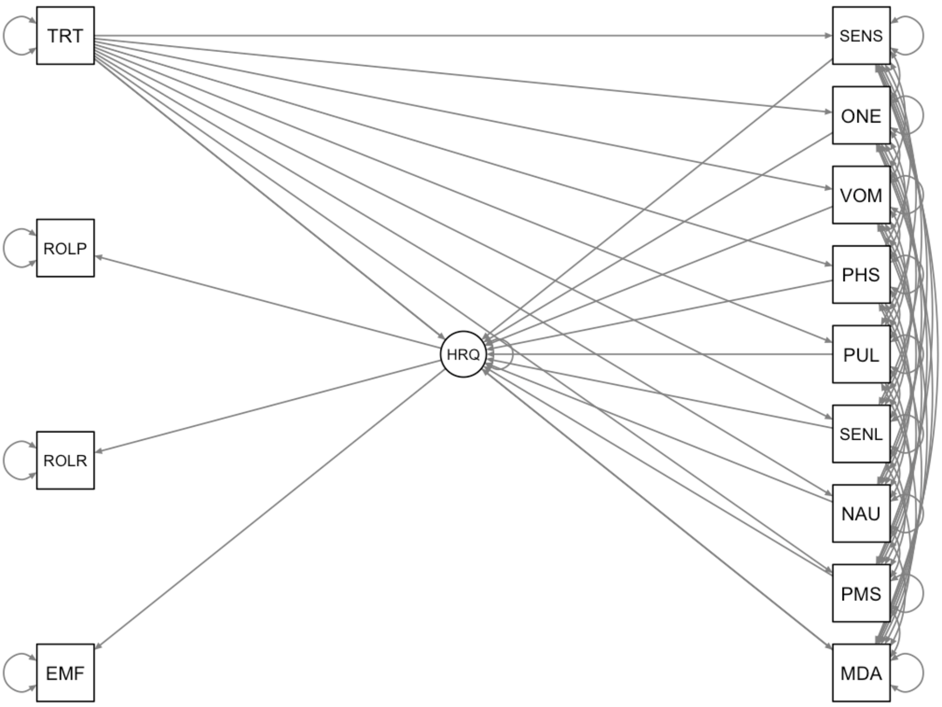

EMF, MSQ Emotional Function; HRQ, health-related quality of life; MDA, monthly migraine days; NAU, nausea; ONE, one-sidedness; PHS, aggravation by physical activity; PMS, migraine severity; PUL, pulsating/throbbing headache; ROLP, MSQ Role Function-Preventive; ROLR, MSQ Role Function-Restrictive; SENL, sensitivity to light; SENS, sensitivity to sound; TRT, treatment (direct effect); VOM, vomiting.

Table S2.D. Model D fit statistics

Number of parameters	70
Degrees of freedom	20
Chi square statistic	31.832
Comparative Fit Index	0.997
RMSEA	0.027
SRMR	0.009
AIC	31,619

AIC, Akaike information criterion; RMSEA, root mean square error of approximation; SRMR, standardized root mean square residual.

Table S3.D. Model D latent variable

Left-hand side variable	Right-hand side variable	Estimate	SE	Z-score	p-value	std.all
HRQoL						
	MSQ-EF	1.000	0.000			0.784
	MSQ-RR	1.056	0.037	28.877	<0.001	0.921
	MSQ-RP	1.012	0.036	27.904	<0.001	0.879

HRQoL, health-related quality of life; MSQ, Migraine-Specific Quality of Life Questionnaire; MSQ-EF, MSQ Emotional Function; MSQ-RP, MSQ Role Function-Preventive; MSQ-RR, MSQ Role Function-Restrictive; SE, standard error.

Table S4.D. Model D regressions

Left-hand side variable	Right-hand side variable	Estimate	SE	Z-score	p-value	std.all
HRQoL	MMDs	-1.429	0.135	-10.559	<0.001	-0.356
HRQoL	Severe migraine	-0.099	0.027	-3.656	<0.001	-0.132
HRQoL	Nausea	-8.740	2.477	-3.529	<0.001	-0.128
HRQoL	Light sensitivity	-3.320	3.188	-1.041	0.298	-0.043
HRQoL	Pulsating/throbbing	-4.605	2.888	-1.594	0.111	-0.052
HRQoL	Aggravation by phys. act.	-3.589	2.718	-1.320	0.187	-0.045
HRQoL	Vomiting	-4.388	3.401	-1.290	0.197	-0.042
HRQoL	One-sidedness	-2.049	2.863	-0.716	0.474	-0.023
HRQoL	Sound sensitivity	-2.230	3.210	-0.695	0.487	-0.029
MMDs	Treatment (direct effect)	-3.030	0.332	-9.116	<0.001	-0.301
Severe migraine	Treatment (direct effect)	-11.117	1.826	-6.089	<0.001	-0.206
Nausea	Treatment (direct effect)	-0.114	0.020	-5.643	<0.001	-0.192
Light sensitivity	Treatment (direct effect)	-0.078	0.018	-4.378	<0.001	-0.150
Pulsating/throbbing	Treatment (direct effect)	-0.049	0.016	-3.081	0.002	-0.106
Aggravation by phys. act.	Treatment (direct effect)	-0.050	0.017	-2.874	0.004	-0.099
Vomiting	Treatment (direct effect)	-0.032	0.013	-2.352	0.019	-0.081
One-sidedness	Treatment (direct effect)	-0.065	0.015	-4.271	<0.001	-0.146
Sound sensitivity	Treatment (direct effect)	-0.084	0.018	-4.707	<0.001	-0.161
HRQoL	Treatment (direct effect)	5.040	1.296	3.889	<0.001	0.125

HRQoL, health-related quality of life; MMDs, monthly migraine days; Phys. act., physical activity; SE, standard error.

Table S5.D. Model D covariances

Left-hand side variable	Right-hand side variable	Estimate	SE	Z-score	p-value	std.all
MMDs	Severe migraine	23.140	4.036	5.734	<0.001	0.203
MMDs	Nausea	0.146	0.044	3.321	<0.001	0.116
Severe migraine	Nausea	2.770	0.259	10.712	<0.001	0.400
MMDs	Light sensitivity	0.138	0.039	3.540	<0.001	0.124
Severe migraine	Light sensitivity	1.666	0.220	7.574	<0.001	0.272
Nausea	Light sensitivity	0.025	0.003	10.064	<0.001	0.372
MMDs	Pulsating/throbbing	0.112	0.034	3.242	0.001	0.113
Severe migraine	Pulsating/throbbing	0.986	0.191	5.157	<0.001	0.182
Nausea	Pulsating/throbbing	0.016	0.002	7.295	<0.001	0.261
Light sensitivity	Pulsating/throbbing	0.014	0.002	7.408	<0.001	0.266
MMDs	Aggravation by phys. act.	0.118	0.038	3.116	0.002	0.109
Severe migraine	Aggravation by phys. act.	1.802	0.216	8.360	<0.001	0.303
Nausea	Aggravation by phys. act.	0.016	0.002	6.681	<0.001	0.238
Light sensitivity	Aggravation by phys. act.	0.018	0.002	8.739	<0.001	0.318
Pulsating/throbbing	Aggravation by phys. act.	0.014	0.002	7.322	<0.001	0.262
MMDs	Vomiting	0.065	0.029	2.206	0.027	0.077
Severe migraine	Vomiting	1.652	0.170	9.693	<0.001	0.357
Nausea	Vomiting	0.016	0.002	8.816	<0.001	0.321
Light sensitivity	Vomiting	0.008	0.002	5.165	<0.001	0.182
Pulsating/throbbing	Vomiting	0.004	0.001	3.186	0.001	0.111
Aggravation by phys. act.	Vomiting	0.008	0.002	5.342	<0.001	0.188
MMDs	One-sidedness	0.089	0.033	2.680	0.007	0.093
Severe migraine	One-sidedness	0.143	0.181	0.792	0.428	0.027
Nausea	One-sidedness	0.012	0.002	5.782	<0.001	0.204
Light sensitivity	One-sidedness	0.008	0.002	4.558	<0.001	0.160
Pulsating/throbbing	One-sidedness	0.008	0.002	5.190	<0.001	0.183
Aggravation by phys. act.	One-sidedness	0.007	0.002	3.931	<0.001	0.137
Vomiting	One-sidedness	0.000	0.001	0.319	0.749	0.011
MMDs	Sound sensitivity	0.185	0.039	4.735	<0.001	0.166
Severe migraine	Sound sensitivity	1.431	0.218	6.575	<0.001	0.234
Nausea	Sound sensitivity	0.019	0.002	7.967	<0.001	0.287
Light sensitivity	Sound sensitivity	0.039	0.002	15.627	<0.001	0.644
Pulsating/throbbing	Sound sensitivity	0.017	0.002	8.870	<0.001	0.323
Aggravation by phys. act.	Sound sensitivity	0.022	0.002	10.086	<0.001	0.373
Vomiting	Sound sensitivity	0.005	0.002	3.342	<0.001	0.117
One-sidedness	Sound sensitivity	0.006	0.002	3.376	<0.001	0.118
MMDs	Aura	0.102	0.036	2.866	0.004	0.100
Severe migraine	Aura	0.485	0.195	2.489	0.013	0.087
Nausea	Aura	0.010	0.002	4.766	<0.001	0.167
Light sensitivity	Aura	0.008	0.002	4.409	<0.001	0.155
Pulsating/throbbing	Aura	0.006	0.002	3.412	<0.001	0.119
Aggravation by phys. act.	Aura	0.003	0.002	1.392	0.164	0.048
Vomiting	Aura	0.007	0.001	5.066	<0.001	0.178
One-sidedness	Aura	0.003	0.002	1.761	0.078	0.061
Sound sensitivity	Aura	0.008	0.002	4.155	<0.001	0.145

MMDs, monthly migraine days; Phys. act., physical activity; SE, standard error.

Table S6.D. Model D variances

Left-hand side variable	Estimate	SE	Z-score	p-value	std.all
MSQ-EF	230.750	13.395	17.226	<0.001	0.385
MSQ-RR	73.746	8.182	9.013	<0.001	0.152
MSQ-RP	111.174	8.746	12.712	<0.001	0.228
MMDs	20.783	1.018	20.408	<0.001	0.909
Severe migraine	626.976	30.722	20.408	<0.001	0.957
Nausea	0.077	0.004	20.408	<0.001	0.963
Light sensitivity	0.060	0.003	20.408	<0.001	0.978
Pulsating/throbbing	0.047	0.002	20.408	<0.001	0.989
Aggravation by phys. act.	0.057	0.003	20.408	<0.001	0.990
Vomiting	0.034	0.002	20.408	<0.001	0.993
One-sidedness	0.044	0.002	20.408	<0.001	0.979
Sound sensitivity	0.060	0.003	20.408	<0.001	0.974
HRQoL	237.396	18.477	12.848	<0.001	0.645
Treatment (direct effect)	0.226	0.000			1.000

HRQoL, health-related quality of life; MMDs, monthly migraine days; MSQ, Migraine-Specific Quality of Life Questionnaire; MSQ-EF, MSQ Emotional Function; MSQ-RP, MSQ Role Function-Preventive; MSQ-RR, MSQ Role Function-Restrictive; Phys. act., physical activity; SE, standard error.

Model Details: Model E

Model E includes the three domains of the Migraine-Specific Quality of Life Questionnaire (MSQ) as measures of health-related quality of life (HRQoL), and monthly migraine days (MMDs), severity, nausea, sensitivity to light, pulsating/throbbing headache, aggravation by physical activity, vomiting, and one-sidedness as mediators.

Figure S1.E. Model E structure

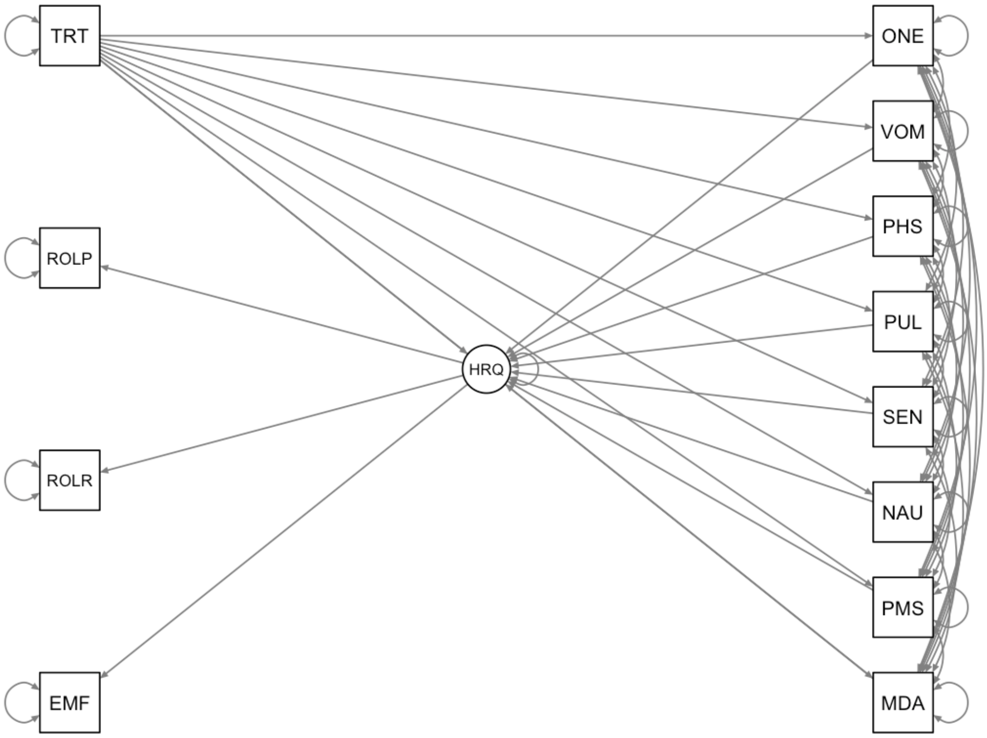

EMF, MSQ Emotional Function; HRQ, health-related quality of life; MDA, monthly migraine days; NAU, nausea; ONE, one-sidedness; PHS, aggravation by physical activity; PMS, migraine severity; PUL, pulsating/throbbing headache; ROLP, MSQ Role Function-Preventive; ROLR, MSQ Role Function-Restrictive; SEN, sensitivity to light; TRT, treatment (direct effect); VOM, vomiting.

Table S2.E. Model E fit statistics

Number of parameters	59
Degrees of freedom	18
Chi square statistic	30.861
Comparative Fit Index	0.996
RMSEA	0.029
SRMR	0.010
AIC	32,108

AIC, Akaike information criterion; RMSEA, root mean square error of approximation; SRMR, standardized root mean square residual.

Table S3.E. Model E latent variable

Left-hand side variable	Right-hand side variable	Estimate	SE	Z-score	p-value	std.all
HRQoL						
	MSQ-EF	1.000	0.000			0.784
	MSQ-RR	1.056	0.037	28.872	<0.001	0.921
	MSQ-RP	1.012	0.036	27.901	<0.001	0.879

HRQoL, health-related quality of life; MSQ, Migraine-Specific Quality of Life Questionnaire; MSQ-EF, MSQ Emotional Function; MSQ-RP, MSQ Role Function-Preventive; MSQ-RR, MSQ Role Function-Restrictive; SE, standard error.

Table S4.E. Model E regressions

Left-hand side variable	Right-hand side variable	Estimate	SE	Z-score	p-value	std.all
HRQoL	MMDs	-1.437	0.135	-10.651	<0.001	-0.358
HRQoL	Severe migraine	-0.099	0.027	-3.659	<0.001	-0.132
HRQoL	Nausea	-8.770	2.477	-3.541	<0.001	-0.129
HRQoL	Light sensitivity	-4.555	2.651	-1.718	0.086	-0.059
HRQoL	Pulsating/throbbing	-4.930	2.851	-1.729	0.084	-0.056
HRQoL	Aggravation by phys. act.	-3.957	2.667	-1.484	0.138	-0.049
HRQoL	Vomiting	-4.275	3.397	-1.259	0.208	-0.041
HRQoL	One-sidedness	-1.978	2.862	-0.691	0.489	-0.022
MMDs	Treatment (direct effect)	-3.030	0.332	-9.116	<0.001	-0.301
Severe migraine	Treatment (direct effect)	-11.117	1.826	-6.089	<0.001	-0.206
Nausea	Treatment (direct effect)	-0.114	0.020	-5.643	<0.001	-0.192
Light sensitivity	Treatment (direct effect)	-0.078	0.018	-4.378	<0.001	-0.150
Pulsating/throbbing	Treatment (direct effect)	-0.049	0.016	-3.081	0.002	-0.106
Aggravation by phys. act.	Treatment (direct effect)	-0.050	0.017	-2.874	0.004	-0.099
Vomiting	Treatment (direct effect)	-0.032	0.013	-2.351	0.019	-0.081
One-sidedness	Treatment (direct effect)	-0.065	0.015	-4.271	<0.001	-0.146
HRQoL	Treatment (direct effect)	5.074	1.295	3.917	<0.001	0.126

HRQoL, health-related quality of life; MMDs, monthly migraine days; Phys. act., physical activity; SE, standard error.

Table S5.E. Model E covariances

Left-hand side variable	Right-hand side variable	Estimate	SE	Z-score	p-value	std.all
MMDs	Severe migraine	23.139	4.036	5.734	<0.001	0.203
MMDs	Nausea	0.146	0.044	3.321	<0.001	0.116
Severe migraine	Nausea	2.770	0.259	10.712	<0.001	0.400
MMDs	Light sensitivity	0.138	0.039	3.540	<0.001	0.124
Severe migraine	Light sensitivity	1.666	0.220	7.574	<0.001	0.272
Nausea	Light sensitivity	0.025	0.003	10.064	<0.001	0.372
MMDs	Pulsating/throbbing	0.112	0.034	3.242	0.001	0.113
Severe migraine	Pulsating/throbbing	0.986	0.191	5.157	<0.001	0.182
Nausea	Pulsating/throbbing	0.016	0.002	7.295	<0.001	0.261
Light sensitivity	Pulsating/throbbing	0.014	0.002	7.408	<0.001	0.266
MMDs	Aggravation by phys. act.	0.118	0.038	3.116	0.002	0.109
Severe migraine	Aggravation by phys. act.	1.802	0.216	8.360	<0.001	0.303
Nausea	Aggravation by phys. act.	0.016	0.002	6.681	<0.001	0.238
Light sensitivity	Aggravation by phys. act.	0.018	0.002	8.739	<0.001	0.318
Pulsating/throbbing	Aggravation by phys. act.	0.014	0.002	7.322	<0.001	0.262
MMDs	Vomiting	0.065	0.029	2.206	0.027	0.077
Severe migraine	Vomiting	1.652	0.170	9.693	<0.001	0.357
Nausea	Vomiting	0.016	0.002	8.816	<0.001	0.321
Light sensitivity	Vomiting	0.008	0.002	5.165	<0.001	0.182
Pulsating/throbbing	Vomiting	0.004	0.001	3.186	0.001	0.111
Aggravation by phys. act.	Vomiting	0.008	0.002	5.342	<0.001	0.188
MMDs	One-sidedness	0.089	0.033	2.680	0.007	0.093
Severe migraine	One-sidedness	0.143	0.181	0.792	0.428	0.027
Nausea	One-sidedness	0.012	0.002	5.782	<0.001	0.204
Light sensitivity	One-sidedness	0.008	0.002	4.558	<0.001	0.160
Pulsating/throbbing	One-sidedness	0.008	0.002	5.190	<0.001	0.183
Aggravation by phys. act.	One-sidedness	0.007	0.002	3.931	<0.001	0.137
Vomiting	One-sidedness	0.000	0.001	0.319	0.749	0.011

MMDs, monthly migraine days; Phys. act., physical activity.

Table S6.E. Model E variances

Left-hand side variable	Estimate	SE	Z-score	p-value	std.all
MSQ-EF	230.830	13.398	17.228	<0.001	0.386
MSQ-RR	73.724	8.184	9.009	<0.001	0.152
MSQ-RP	111.149	8.746	12.708	<0.001	0.228
MMDs	20.783	1.018	20.408	<0.001	0.909
Severe migraine	626.976	30.722	20.408	<0.001	0.957
Nausea	0.077	0.004	20.408	<0.001	0.963
Light sensitivity	0.060	0.003	20.408	<0.001	0.978
Pulsating/throbbing	0.047	0.002	20.408	<0.001	0.989
Aggravation by phys. act.	0.057	0.003	20.408	<0.001	0.990
Vomiting	0.034	0.002	20.408	<0.001	0.993
One-sidedness	0.044	0.002	20.408	<0.001	0.979
HRQoL	237.503	18.487	12.847	<0.001	0.646
Treatment (direct effect)	0.226	0.000			1.000

HRQoL, health-related quality of life; MMDs, monthly migraine days; MSQ, Migraine-Specific Quality of Life Questionnaire; MSQ-EF, MSQ Emotional Function; MSQ-RP, MSQ Role Function-Preventive; MSQ-RR, MSQ Role Function-Restrictive; Phys. act., physical activity; SE, standard error.

Model Details: Model F

Model F includes the three domains of the Migraine-Specific Quality of Life Questionnaire (MSQ) as measures of health-related quality of life (HRQoL), and monthly migraine days (MMDs), severity, nausea, sensitivity to light, pulsating/throbbing headache, aggravation by physical activity, and vomiting as mediators.

Figure S1.F. Model F structure

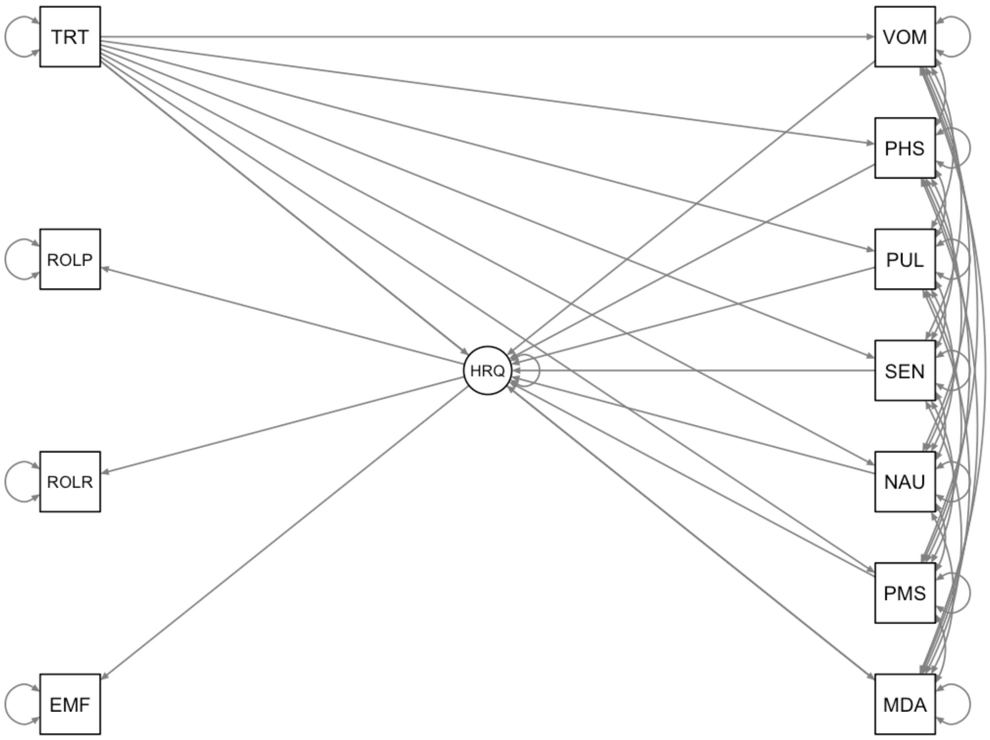

EMF, MSQ Emotional Function; HRQ, health-related quality of life; MDA, monthly migraine days; NAU, nausea; PHS, aggravation by physical activity; PMS, migraine severity; PUL, pulsating/throbbing headache; ROLP, MSQ Role Function-Preventive; ROLR, MSQ Role Function-Restrictive; SEN, sensitivity to light; TRT, treatment (direct effect); VOM, vomiting.

Table S2.F. Model F fit statistics

Number of parameters	49
Degrees of freedom	16
Chi square statistic	27.697
Comparative Fit Index	0.996
RMSEA	0.030
SRMR	0.010
AIC	32,407

AIC, Akaike information criterion; RMSEA, root mean square error of approximation; SRMR, standardized root mean square residual.

Table S3.F. Model F latent variable

Left-hand side variable	Right-hand side variable	Estimate	SE	Z-score	p-value	std.all
HRQoL						
	MSQ-EF	1.000	0.000			0.784
	MSQ-RR	1.056	0.037	28.877	<0.001	0.921
	MSQ-RP	1.012	0.036	27.911	<0.001	0.879

HRQoL, health-related quality of life; MSQ, Migraine-Specific Quality of Life Questionnaire; MSQ-EF, MSQ Emotional Function; MSQ-RP, MSQ Role Function-Preventive; MSQ-RR, MSQ Role Function-Restrictive; SE, standard error.

Table S4.F. Model F regressions

Left-hand side variable	Right-hand side variable	Estimate	SE	Z-score	p-value	std.all
HRQoL	MMDs	-1.443	0.135	-10.713	<0.001	-0.360
HRQoL	Severe migraine	-0.097	0.027	-3.611	<0.001	-0.130
HRQoL	Nausea	-9.050	2.447	-3.698	<0.001	-0.133
HRQoL	Light sensitivity	-4.663	2.647	-1.761	0.078	-0.060
HRQoL	Pulsating/throbbing	-5.148	2.836	-1.816	0.069	-0.059
HRQoL	Aggravation by phys. act.	-4.093	2.661	-1.538	0.124	-0.051
HRQoL	Vomiting	-4.146	3.394	-1.222	0.222	-0.040
MMDs	Treatment (direct effect)	-3.030	0.332	-9.116	<0.001	-0.301
Severe migraine	Treatment (direct effect)	-11.117	1.826	-6.089	<0.001	-0.206
Nausea	Treatment (direct effect)	-0.114	0.020	-5.643	<0.001	-0.192
Light sensitivity	Treatment (direct effect)	-0.078	0.018	-4.378	<0.001	-0.150
Pulsating/throbbing	Treatment (direct effect)	-0.049	0.016	-3.081	0.002	-0.106
Aggravation by phys. act.	Treatment (direct effect)	-0.050	0.017	-2.874	0.004	-0.099
Vomiting	Treatment (direct effect)	-0.032	0.013	-2.352	0.019	-0.081
HRQoL	Treatment (direct effect)	5.151	1.292	3.988	<0.001	0.128

HRQoL, health-related quality of life; MMDs, monthly migraine days; Phys. act., physical activity; SE, standard error.

Table S5.F. Model F covariances

Left-hand side variable	Right-hand side variable	Estimate	SE	Z-score	p-value	std.all
MMDs	Severe migraine	23.139	4.036	5.734	<0.001	0.203
MMDs	Nausea	0.146	0.044	3.321	<0.001	0.116
Severe migraine	Nausea	2.770	0.259	10.712	<0.001	0.400
MMDs	Light sensitivity	0.138	0.039	3.540	<0.001	0.124
Severe migraine	Light sensitivity	1.666	0.220	7.574	<0.001	0.272
Nausea	Light sensitivity	0.025	0.003	10.064	<0.001	0.372
MMDs	Pulsating/throbbing	0.112	0.034	3.242	0.001	0.113
Severe migraine	Pulsating/throbbing	0.986	0.191	5.157	<0.001	0.182
Nausea	Pulsating/throbbing	0.016	0.002	7.295	<0.001	0.261
Light sensitivity	Pulsating/throbbing	0.014	0.002	7.408	<0.001	0.266
MMDs	Aggravation by phys. act.	0.118	0.038	3.116	0.002	0.109
Severe migraine	Aggravation by phys. act.	1.802	0.216	8.360	<0.001	0.303
Nausea	Aggravation by phys. act.	0.016	0.002	6.681	<0.001	0.238
Light sensitivity	Aggravation by phys. act.	0.018	0.002	8.739	<0.001	0.318
Pulsating/throbbing	Aggravation by phys. act.	0.014	0.002	7.322	<0.001	0.262
MMDs	Vomiting	0.065	0.029	2.206	0.027	0.077
Severe migraine	Vomiting	1.652	0.170	9.693	<0.001	0.357
Nausea	Vomiting	0.016	0.002	8.816	<0.001	0.321
Light sensitivity	Vomiting	0.008	0.002	5.165	<0.001	0.182
Pulsating/throbbing	Vomiting	0.004	0.001	3.186	0.001	0.111
Aggravation by phys. act.	Vomiting	0.008	0.002	5.342	<0.001	0.188

MMDs, monthly migraine days; Phys. act., physical activity.

Table S6.F. Model F variances

Left-hand side variable	Estimate	SE	Z-score	p-value	std.all
MSQ-EF	230.655	13.393	17.222	<0.001	0.385
MSQ-RR	73.885	8.186	9.025	<0.001	0.153
MSQ-RP	111.082	8.746	12.701	<0.001	0.228
MMDs	20.783	1.018	20.408	<0.001	0.909
Severe migraine	626.976	30.722	20.408	<0.001	0.957
Nausea	0.077	0.004	20.408	<0.001	0.963
Light sensitivity	0.060	0.003	20.408	<0.001	0.978
Pulsating/throbbing	0.047	0.002	20.408	<0.001	0.989
Aggravation by phys. act.	0.057	0.003	20.408	<0.001	0.990
Vomiting	0.034	0.002	20.408	<0.001	0.993
HRQoL	237.769	18.502	12.851	<0.001	0.646
Treatment (direct effect)	0.226	0.000			1.000

HRQoL, health-related quality of life; MMDs, monthly migraine days; MSQ, Migraine-Specific Quality of Life Questionnaire; MSQ-EF, MSQ Emotional Function; MSQ-RP, MSQ Role Function-Preventive; MSQ-RR, MSQ Role Function-Restrictive; Phys. act., physical activity; SE, standard error.

Model Details: Model G

Model G includes the three domains of the Migraine-Specific Quality of Life Questionnaire (MSQ) as measures of health-related quality of life (HRQoL), and monthly migraine days (MMDs), severity, nausea, sensitivity to light, pulsating/throbbing headache, and aggravation by physical activity as mediators.

Figure S1.G. Model G structure

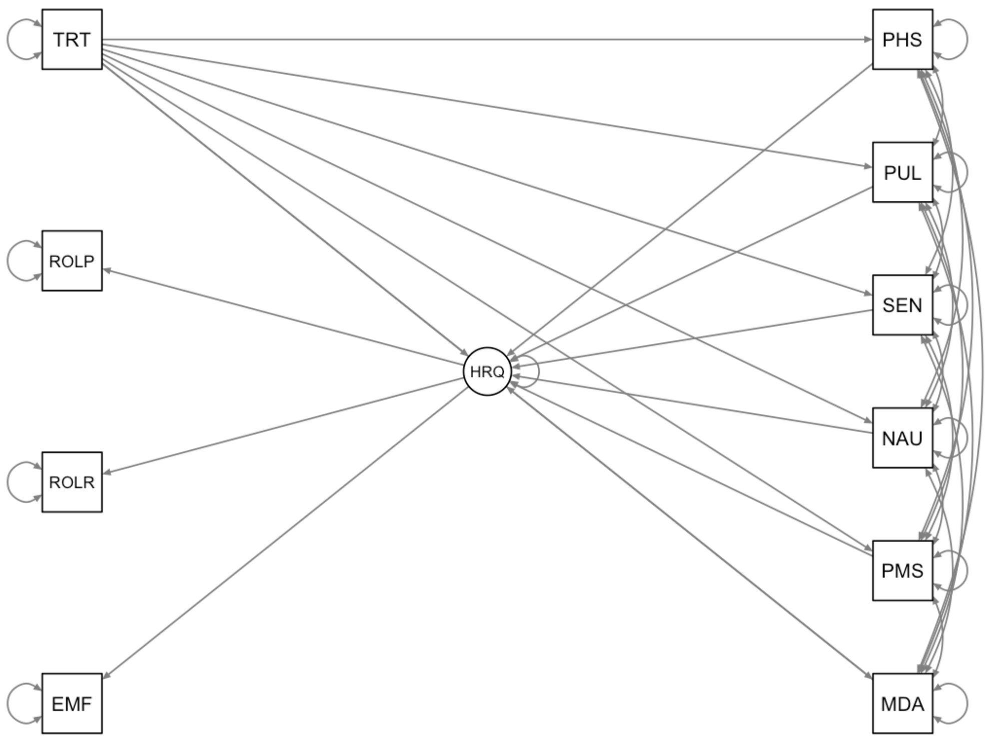

EMF, MSQ Emotional Function; HRQ, health-related quality of life; MDA, monthly migraine days; NAU, nausea; PHS, aggravation by physical activity; PMS, migraine severity; PUL, pulsating/throbbing headache; ROLP, MSQ Role Function-Preventive; ROLR, MSQ Role Function-Restrictive; SEN, sensitivity to light; TRT, treatment (direct effect).

Table S2.G. Model G fit statistics

Number of parameters	40
Degrees of freedom	14
Chi square statistic	17.643
Comparative Fit Index	0.999
RMSEA	0.018
SRMR	0.009
AIC	32,992

AIC, Akaike information criterion; RMSEA, root mean square error of approximation; SRMR, standardized root mean square residual.

Table S3.G. Model G latent variable

Left-hand side variable	Right-hand side variable	Estimate	SE	Z-score	p-value	std.all
HRQoL						
	MSQ-EF	1.000	0.000			0.783
	MSQ-RR	1.057	0.037	28.861	<0.001	0.921
	MSQ-RP	1.012	0.036	27.869	<0.001	0.878

HRQoL, health-related quality of life; MSQ, Migraine-Specific Quality of Life Questionnaire; MSQ-EF, MSQ Emotional Function; MSQ-RP, MSQ Role Function-Preventive; MSQ-RR, MSQ Role Function-Restrictive; SE, standard error.

Table S4.G. Model G regressions

Left-hand side variable	Right-hand side variable	Estimate	SE	Z-score	p-value	std.all
HRQoL	MMDs	-1.441	0.135	-10.697	<0.001	-0.359
HRQoL	Severe migraine	-0.105	0.026	-4.012	<0.001	-0.140
HRQoL	Nausea	-9.579	2.407	-3.980	<0.001	-0.141
HRQoL	Light sensitivity	-4.742	2.647	-1.792	0.073	-0.061
HRQoL	Pulsating/throbbing	-5.119	2.835	-1.805	0.071	-0.058
HRQoL	Aggravation by phys. act.	-4.284	2.657	-1.612	0.107	-0.053
MMDs	Treatment (direct effect)	-3.030	0.332	-9.116	<0.001	-0.301
Severe migraine	Treatment (direct effect)	-11.117	1.826	-6.089	<0.001	-0.206
Nausea	Treatment (direct effect)	-0.114	0.020	-5.643	<0.001	-0.192
Light sensitivity	Treatment (direct effect)	-0.078	0.018	-4.378	<0.001	-0.150
Pulsating/throbbing	Treatment (direct effect)	-0.049	0.016	-3.081	0.002	-0.106
Aggravation by phys. act.	Treatment (direct effect)	-0.050	0.017	-2.874	0.004	-0.099
HRQoL	Treatment (direct effect)	5.119	1.291	3.964	<0.001	0.127

HRQoL, health-related quality of life; MMDs, monthly migraine days; Phys. act., physical activity; SE, standard error.

Table S5.G. Model G covariances

Left-hand side variable	Right-hand side variable	Estimate	SE	Z-score	p-value	std.all
MMDs	Severe migraine	23.139	4.036	5.734	<0.001	0.203
MMDs	Nausea	0.146	0.044	3.321	<0.001	0.116
Severe migraine	Nausea	2.770	0.259	10.712	<0.001	0.400
MMDs	Light sensitivity	0.138	0.039	3.540	<0.001	0.124
Severe migraine	Light sensitivity	1.666	0.220	7.574	<0.001	0.272
Nausea	Light sensitivity	0.025	0.003	10.064	<0.001	0.372
MMDs	Pulsating/throbbing	0.112	0.034	3.242	0.001	0.113
Severe migraine	Pulsating/throbbing	0.986	0.191	5.157	<0.001	0.182
Nausea	Pulsating/throbbing	0.016	0.002	7.295	<0.001	0.261
Light sensitivity	Pulsating/throbbing	0.014	0.002	7.408	<0.001	0.266
MMDs	Aggravation by phys. act.	0.118	0.038	3.116	0.002	0.109
Severe migraine	Aggravation by phys. act.	1.802	0.216	8.360	<0.001	0.303
Nausea	Aggravation by phys. act.	0.016	0.002	6.681	<0.001	0.238
Light sensitivity	Aggravation by phys. act.	0.018	0.002	8.739	<0.001	0.318
Pulsating/throbbing	Aggravation by phys. act.	0.014	0.002	7.322	<0.001	0.262

MMDs, monthly migraine days; Phys. act., physical activity; SE, standard error.

Table S6.G. Model G variances

Left-hand side variable	Estimate	SE	Z-score	p-value	std.all
MSQ-EF	231.190	13.411	17.239	<0.001	0.386
MSQ-RR	73.127	8.185	8.934	<0.001	0.151
MSQ-RP	111.578	8.758	12.740	<0.001	0.229
MMDs	20.783	1.018	20.408	<0.001	0.909
Severe migraine	626.976	30.722	20.408	<0.001	0.957
Nausea	0.077	0.004	20.408	<0.001	0.963
Light sensitivity	0.060	0.003	20.408	<0.001	0.978
Pulsating/throbbing	0.047	0.002	20.408	<0.001	0.989
Aggravation by phys. act.	0.057	0.003	20.408	<0.001	0.990
HRQoL	237.918	18.526	12.842	<0.001	0.647
Treatment (direct effect)	0.226	0.000			1.000

HRQoL, health-related quality of life; MMDs, monthly migraine days; MSQ, Migraine-Specific Quality of Life Questionnaire; MSQ-EF, MSQ Emotional Function; MSQ-RP, MSQ Role Function-Preventive; MSQ-RR, MSQ Role Function-Restrictive; Phys. act., physical activity; SE, standard error.

Model Details: Model 1

Final Model 1 includes the three domains of the Migraine-Specific Quality of Life Questionnaire (MSQ) as measures of health-related quality of life (HRQoL), and monthly migraine days (MMDs), severity, nausea, sensitivity to light and pulsating/throbbing headache as mediators.

Figure S1.1. Model 1 structure

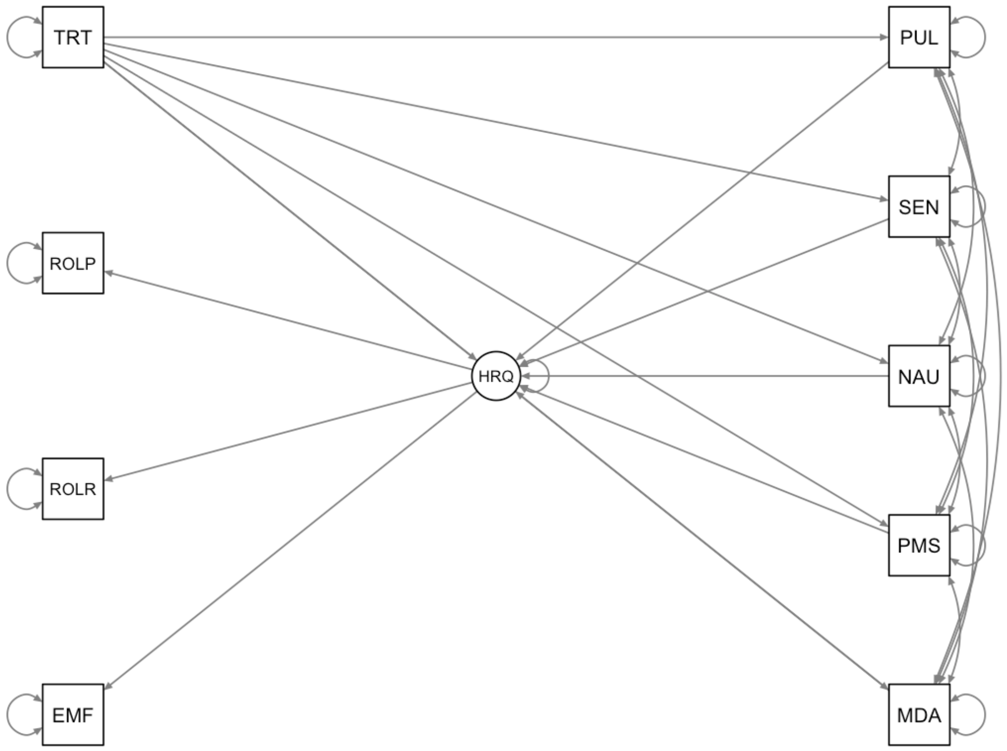

EMF, MSQ Emotional Function; HRQ, health-related quality of life; MDA, monthly migraine days; NAU, nausea; PMS, migraine severity; PUL, pulsating/throbbing headache; ROLP, MSQ Role Function-Preventive; ROLR, MSQ Role Function-Restrictive; SEN, sensitivity to light; TRT, treatment (direct effect).

Table S2.1. Model 1 fit statistics

Number of parameters	32
Degrees of freedom	12
Chi square statistic	16.602
Comparative Fit Index	0.998
RMSEA	0.021
SRMR	0.009
AIC	33,171

AIC, Akaike information criterion; RMSEA, root mean square error of approximation; SRMR, standardized root mean square residual.

Table S3.1. Model 1 latent variable

Left-hand side variable	Right-hand side variable	Estimate	SE	Z-score	p-value	std.all
HRQoL						
	MSQ-EF	1.000	0.000			0.784
	MSQ-RR	1.057	0.037	28.865	<0.001	0.921
	MSQ-RP	1.012	0.036	27.881	<0.001	0.878

HRQoL, health-related quality of life; MSQ, Migraine-Specific Quality of Life Questionnaire; MSQ-EF, MSQ Emotional Function; MSQ-RP, MSQ Role Function-Preventive; MSQ-RR, MSQ Role Function-Restrictive; SE, standard error.

Table S4.1. Model 1 regressions

Left-hand side variable	Right-hand side variable	Estimate	SE	Z-score	p-value	std.all
HRQoL	MMDs	-1.446	0.135	-10.716	<0.001	-0.361
HRQoL	Severe migraine	-0.113	0.026	-4.389	<0.001	-0.151
HRQoL	Nausea	-9.728	2.410	-4.036	<0.001	-0.143
HRQoL	Light sensitivity	-5.592	2.600	-2.151	0.031	-0.072
HRQoL	Pulsating/throbbing	-5.869	2.803	-2.094	0.036	-0.067
MMDs	Treatment (direct effect)	-3.030	0.332	-9.116	<0.001	-0.301
Severe migraine	Treatment (direct effect)	-11.117	1.826	-6.089	<0.001	-0.206
Nausea	Treatment (direct effect)	-0.114	0.020	-5.643	<0.001	-0.192
Light sensitivity	Treatment (direct effect)	-0.078	0.018	-4.378	<0.001	-0.150
Pulsating/throbbing	Treatment (direct effect)	-0.049	0.016	-3.081	0.002	-0.106
HRQoL	Treatment (direct effect)	5.111	1.294	3.950	<0.001	0.127

HRQoL, health-related quality of life; MMDs, monthly migraine days; SE, standard error.

Table S5.1. Model 1 covariances

Left-hand side variable	Right-hand side variable	Estimate	SE	Z-score	p-value	std.all
MMDs	Severe migraine	23.139	4.036	5.734	<0.001	0.203
MMDs	Nausea	0.146	0.044	3.321	<0.001	0.116
Severe migraine	Nausea	2.770	0.259	10.712	<0.001	0.400
MMDs	Light sensitivity	0.138	0.039	3.540	<0.001	0.124
Severe migraine	Light sensitivity	1.666	0.220	7.574	<0.001	0.272
Nausea	Light sensitivity	0.025	0.003	10.064	<0.001	0.372
MMDs	Pulsating/throbbing	0.112	0.034	3.242	0.001	0.113
Severe migraine	Pulsating/throbbing	0.986	0.191	5.157	<0.001	0.182
Nausea	Pulsating/throbbing	0.016	0.002	7.295	<0.001	0.261
Light sensitivity	Pulsating/throbbing	0.014	0.002	7.408	<0.001	0.266

MMDs, monthly migraine days; SE, standard error.

Table S6.1. Model 1 variances

Left-hand side variable	Estimate	SE	Z-score	p-value	std.all
MSQ-EF	230.954	13.405	17.229	<0.001	0.386
MSQ-RR	73.339	8.194	8.950	<0.001	0.152
MSQ-RP	111.492	8.762	12.724	<0.001	0.228
MMDs	20.783	1.018	20.408	<0.001	0.909
Severe migraine	626.976	30.722	20.408	<0.001	0.957
Nausea	0.077	0.004	20.408	<0.001	0.963
Light sensitivity	0.060	0.003	20.408	<0.001	0.978
Pulsating/throbbing	0.047	0.002	20.408	<0.001	0.989
HRQoL	238.918	18.593	12.850	<0.001	0.650
Treatment (direct effect)	0.226	0.000			1.000

HRQoL, health-related quality of life; MMDs, monthly migraine days; MSQ, Migraine-Specific Quality of Life Questionnaire; MSQ-EF, MSQ Emotional Function; MSQ-RP, MSQ Role Function-Preventive; MSQ-RR, MSQ Role Function-Restrictive; SE, standard error.

Model Details: Model 2

Final Model 2 includes the three domains of the Migraine-Specific Quality of Life Questionnaire (MSQ) as measures of health-related quality of life (HRQoL), and monthly migraine days (MMDs) and patient-identified most bothersome symptom (PI-MBS) as mediators.

Figure S1.2. Model 2 structure

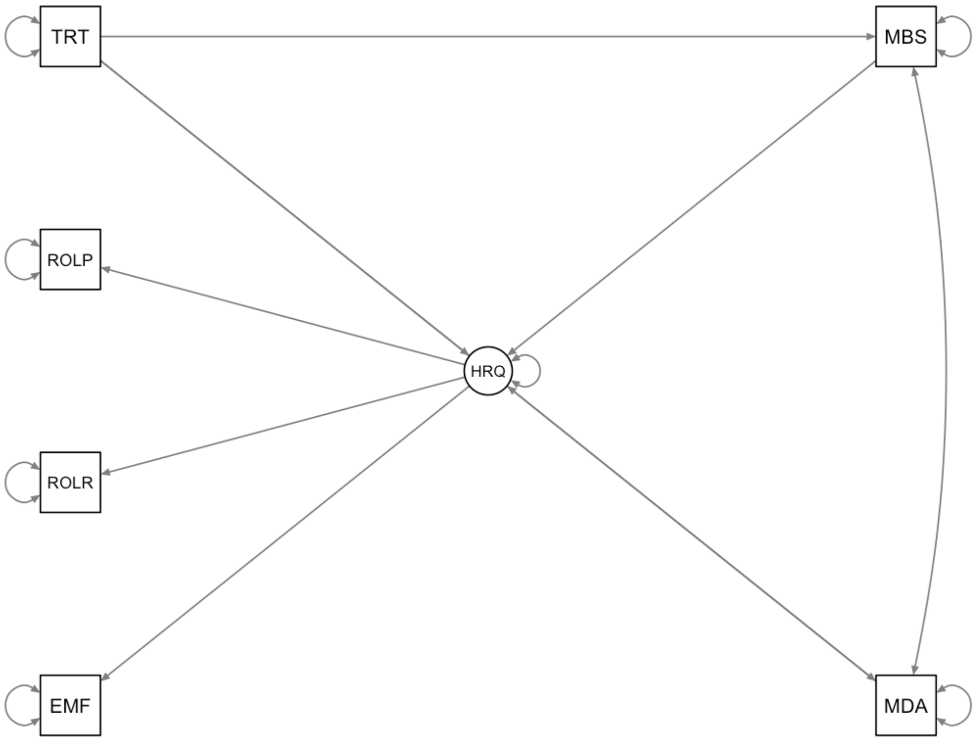

EMF, MSQ Emotional Function; HRQ, health-related quality of life; MDA, monthly migraine days; PI-MBS, patient-identified most bothersome symptom; ROLP, MSQ Role Function-Preventive; ROLR, MSQ Role Function-Restrictive; TRT, treatment (direct effect).

Table S2.2. Model 2 fit statistics

Number of parameters	14
Degrees of freedom	6
Chi square statistic	14.580
Comparative Fit Index	0.996
RMSEA	0.041
SRMR	0.011
AIC	27,814

AIC, Akaike information criterion; RMSEA, root mean square error of approximation; SRMR, standardized root mean square residual.

Table S3.2. Model 2 latent variable

Left-hand side variable	Right-hand side variable	Estimate	SE	Z-score	p-value	std.all
HRQoL						
	MSQ-EF	1.000	0.000			0.781
	MSQ-RR	1.072	0.037	29.193	<0.001	0.931
	MSQ-RP	1.005	0.036	27.579	<0.001	0.869

HRQoL, health-related quality of life; MSQ, Migraine-Specific Quality of Life Questionnaire; MSQ-EF, MSQ Emotional Function; MSQ-RP, MSQ Role Function-Preventive; MSQ-RR, MSQ Role Function-Restrictive; SE, standard error.

Table S4.2. Model 2 regressions

Left-hand side variable	Right-hand side variable	Estimate	SE	Z-score	p-value	std.all
HRQoL	MMDs	-0.718	0.134	-5.373	<0.001	-0.180
HRQoL	PI-MBS	-9.322	0.650	-14.350	<0.001	-0.538
MMDs	Treatment (direct effect)	-3.030	0.332	-9.116	<0.001	-0.301
PI-MBS	Treatment (direct effect)	-0.932	0.074	-12.646	<0.001	-0.401
HRQoL	Treatment (direct effect)	1.667	1.224	1.362	0.173	0.041

HRQoL, health-related quality of life; MMDs, monthly migraine days; PI-MBS, patient-identified most bothersome symptom; SE, standard error.

Table S5.2. Model 2 covariances

Left-hand side variable	Right-hand side variable	Estimate	SE	Z-score	p-value	std.all
MMDs	PI-MBS	2.196	0.177	12.419	<0.001	0.477

MMDs, monthly migraine days; PI-MBS, patient-identified most bothersome symptom; SE, standard error.

Table S6.2. Model 2 variances

Left-hand side variable	Estimate	SE	Z-score	p-value	std.all
MSQ-EF	233.726	13.392	17.453	<0.001	0.390
MSQ-RR	64.350	7.749	8.304	<0.001	0.133
MSQ-RP	119.922	8.680	13.816	<0.001	0.246
MMDs	20.783	1.018	20.408	<0.001	0.909
PI-MBS	1.021	0.050	20.408	<0.001	0.839
HRQoL	200.649	15.806	12.694	<0.001	0.550
Treatment (direct effect)	0.226	0.000			1.000

HRQoL, health-related quality of life; MMDs, monthly migraine days; MSQ, Migraine-Specific Quality of Life Questionnaire; MSQ-EF, MSQ Emotional Function; MSQ-RP, MSQ Role Function-Preventive; MSQ-RR, MSQ Role Function-Restrictive; PI-MBS, patient-identified most bothersome symptom; SE, standard error.

Supplemental Table 7. Estimated coefficients of Final Model 1 separately by dosage of eptinezumab (100 mg vs 300 mg)

			Eptinezumab 100 mg					Eptinezumab 300 mg				
LHS		RHS	Estimate	StdErr	Z-score	p-value	Std.estimate	Estimate	StdErr	Z-score	p-value	Std.estimate
HRQoL	=~	EMFUN	1.000	0.000			0.797	1.000	0.000			0.789
HRQoL	=~	ROLPR	1.043	0.045	23.421	<0.001	0.881	0.950	0.042	22.496	<0.001	0.862
HRQoL	=~	ROLRE	1.062	0.044	24.136	<0.001	0.915	0.990	0.042	23.566	<0.001	0.915
HRQoL	~	MDAYI	-1.420	0.167	-8.495	<0.001	-0.351	-1.505	0.171	-8.803	<0.001	-0.360
HRQoL	~	NAUSEA	-10.478	3.003	-3.490	<0.001	-0.150	-11.888	3.000	-3.963	<0.001	-0.171
HRQoL	~	PMSEV	-0.102	0.031	-3.334	0.001	-0.140	-0.116	0.034	-3.444	0.001	-0.142
HRQoL	~	PULTHR	-5.300	3.555	-1.491	0.136	-0.059	-5.959	3.520	-1.693	0.090	-0.065
HRQoL	~	SENLGT	-4.362	3.217	-1.356	0.175	-0.057	-6.241	3.260	-1.915	0.056	-0.076
HRQoL	~	TRT	5.141	1.487	3.456	0.001	0.138	5.354	1.561	3.431	0.001	0.135
MDAYI	~	TRT	-2.766	0.373	-7.421	<0.001	-0.300	-3.288	0.376	-8.738	<0.001	-0.346
NAUSEA	~	TRT	-0.101	0.022	-4.550	<0.001	-0.189	-0.126	0.023	-5.379	<0.001	-0.221
PMSEV	~	TRT	-11.327	2.120	-5.344	<0.001	-0.221	-10.911	2.001	-5.453	<0.001	-0.224
PULTHR	~	TRT	-0.040	0.017	-2.306	0.021	-0.097	-0.057	0.018	-3.174	0.002	-0.133
SENLGT	~	TRT	-0.082	0.020	-4.081	<0.001	-0.170	-0.074	0.020	-3.671	<0.001	-0.153

EMFUN, MSQ Emotional Function; HRQoL, health-related quality of life; MDAYI, monthly migraine days; MSQ, Migraine-Specific Quality of Life Questionnaire; NAUSEA, nausea; PMSEV, migraine severity; PULTHR, pulsating/throbbing headache; ROLPR, MSQ Role Function-Preventive; ROLRE, MSQ Role Function-Restrictive; SENLGT, sensitivity to light; TRT, treatment (direct effect).

Supplemental Table 8. Mediation analysis with Final Model 1 separately by dosage of eptinezumab (100 mg vs 300 mg)

	Eptinezumab 100 mg					Eptinezumab 300 mg				
	Estimate	StdErr	Z-score	p-value	Std.estimate	Estimate	StdErr	Z-score	p-value	Std.estimate
HRQoL_DIR	5.141	1.487	3.456	0.001	0.138	5.354	1.561	3.431	0.001	0.135
INDIR_HRQoL	6.715	0.944	7.114	<0.001	0.180	8.517	1.081	7.877	<0.001	0.214
HRQoL_MDAYI	3.928	0.703	5.589	<0.001	0.105	4.950	0.798	6.201	<0.001	0.124
HRQoL_NAUSEA	1.060	0.383	2.769	0.006	0.028	1.501	0.470	3.191	0.001	0.038
HRQoL_PMSEV	1.155	0.408	2.829	0.005	0.031	1.264	0.434	2.912	0.004	0.032
HRQoL_PULTHR	0.212	0.170	1.252	0.211	0.006	0.341	0.228	1.494	0.135	0.009
HRQoL_SENLGT	0.360	0.279	1.287	0.198	0.010	0.461	0.272	1.698	0.090	0.012
TOTAL_HRQoL	11.856	1.620	7.318	<0.001	0.318	13.871	1.721	8.058	<0.001	0.349

HRQoL, health-related quality of life; HRQoL_DIR, direct effect on HRQoL; HRQoL_MDAYI, HRQoL effect through monthly migraine days; HRQoL_NAUSEA, HRQoL effect through nausea; HRQoL_PMSEV, HRQoL effect through migraine severity; HRQoL_PULTHR, HRQoL effect through pulsating/throbbing headache; HRQoL_SENLGT, HRQoL effect through sensitivity to light; INDIR_HRQoL, indirect (mediated) effect on HRQoL; TOTAL_HRQoL, total effect on HRQoL.
